# Supplementary material for: SSHeW study protocol: does slip resistant footwear reduce slips among healthcare workers? A randomised controlled trial
Source: BMJ Open. 2018 Nov 15;8(11):e026023. doi: 10.1136/bmjopen-2018-026023 (PMC6252757; doi:10.1136/bmjopen-2018-026023)
Supplement: Author's manuscript [file bmjopen-2018-026023.draft_revisions.pdf]

BMJ Open is committed to open peer review. As part of this commitment we make the peer review history of every article we publish publicly available.

When an article is published we post the peer reviewers' comments and the authors' responses online. We also post the versions of the paper that were used during peer review. These are the versions that the peer review comments apply to.

The versions of the paper that follow are the versions that were submitted during the peer review process. They are not the versions of record or the final published versions. They should not be cited or distributed as the published version of this manuscript.

BMJ Open is an open access journal and the full, final, typeset and author-corrected version of record of the manuscript is available on our site with no access controls, subscription charges or pay-per-view fees (http://bmjopen.bmj.com).

If you have any questions on BMJ Open's open peer review process please email editorial.bmjopen@bmj.com

BMJ Open

The SSHeW study protocol: Does slip resistant footwear reduce slips among healthcare workers? A randomised controlled trial.

Journal:	*BMJ Open*
Manuscript ID	bmjopen-2018-026023
Article Type:	Protocol
Date Submitted by the Author:	15-Aug-2018
Complete List of Authors:	Cockayne, Sarah; UNIVERSITY OF YORK, Department of Health Sciences<br>Fairhurst, Caroline; University of York, Health Sciences<br>Frost, Gillian; Health and Safety Executive<br>Hewitt, Catherine; The University of York, Health Sciences<br>Liddle, Mark ; Health and Safety Executive , Ergonomics & Human Factors<br>Zand, Michael; Health and Safety Executive<br>Iles-Smith , Heather; Leeds Teaching Hospitals NHS Trust<br>Green , Lorraine; Leeds Biomedical Research Centre<br>Cunningham-Burley, Rachel; University of York, Health Sciences<br>Torgerson, David; The University of York,
Keywords:	Slip prevention, slip resistant footwear, randomised controlled trial, short messaging service, national health service

SCHOLARONE™
Manuscripts

The SSHeW study protocol: Does slip resistant footwear reduce slips among healthcare workers? A randomised controlled trial.

Sarah Cockayne[1], Caroline Fairhurst[1], Gillian Frost[2], Catherine Hewitt[1], Mark Liddle[2], Michael Zand[3], Heather Illes-Smith[4], Lorraine Green [4,5], Rachel Cunningham-Burley[1] and David Torgerson[1] on behalf of the SSHeW study

[1] York Trials Unit, Department of Health Sciences, University of York, YO10 5DD, UK

[2] Health and Safety Executive, Buxton, SK17 9JN, UK

[3] Health and Safety Executive, Bootle, L20 7HS, UK

[4] The Leeds Teaching Hospitals NHS Trust, Leeds UK

[5] National Institute for Health Research (NIHR) Leeds Musculoskeletal Biomedical Research Unit, Chapel Allerton Hospital, Leeds, LS7 4SA, UK

$ corresponding author Mrs Sarah Cockayne; University of York, Department of Health Sciences, ARRC Building - Lower Ground Floor, York YO10 5DD. Email sarah.cockayne@york.ac.uk telephone 01904 321736, Fax 01904 321387.

$ SC: sarah.cockayne@york.ac.uk

CF: caroline.fairhurst@york.ac.uk

GF: Gillian.Frost@hse.gov.uk

CH: catherine.hewitt@york.ac.uk

ML: Mark.Liddle@hse.gov.uk

MZ: Michael.Zand@hse.gov.uk

HIS: heather.iles-smith@nhs.net

LG: l.green@leeds.ac.uk

RCB: rachel.cunningham-burley@york.ac.uk

DT: david.torgerson@york.ac.uk

Key words

Slip prevention, slip resistant footwear, randomised controlled trial, National Health Service, short messaging service

Word Count 7,391

For peer review only

For peer review only - http://bmjopen.bmj.com/site/about/guidelines.xhtml

ABSTRACT

Introduction: Slips, trips and falls are common causes of injuries in the workplace. It is estimated that in Great Britain, nearly one million days are taken off work due to these injuries. There is some evidence to suggest this accident burden could be reduced by the use of slip resistant footwear. This protocol describes a multicentre trial investigating the effectiveness and cost-effectiveness of slip resistant footwear to prevent slips in NHS staff working in clinical, general or catering environments.

Methods and analysis: A two-arm, randomised controlled trial conducted within England, with 4,400 NHS staff, aged 18 and above, who adhere to a dress code policy and work in a clinical, catering or general hospital environment. Participants will be randomised 1:1 to the intervention or waiting list control group. The intervention group will be offered a pair of 5-star GRIP rated slip resistant footwear. The control group will be offered the footwear at the end of the trial. The primary outcome is the incidence rate of self-reported slips in the workplace over a 14-week period, as reported via weekly text messages. Secondary outcomes include: time to first slip/fall, proportion of participants who slip and fall over 14 weeks, and incidence rate of falls resulting from and not resulting from a slip in the workplace over 14 weeks. An economic evaluation will assess cost-effectiveness, in terms of cost per quality adjusted life year gained. A nested qualitative study will explore the acceptability of the footwear and compliance.

Ethics and dissemination: This protocol received a favourable ethical opinion from the University of York, Department of Health Sciences Research Governance Committee. The trial results will be published in peer-reviewed journals and at conferences. A summary of the findings will be made available to participants.

Trial registration: Current Controlled Trials ISRCTN33051393, assigned 14/03/2017

For peer review only

For peer review only - http://bmjopen.bmj.com/site/about/guidelines.xhtml

Article Summary

Strengths and limitations of this study

Strengths and limitations of this study

- This is the first randomised controlled trial assessing the effectiveness and cost effectiveness of slip resistant footwear for slip prevention in the UK.
- The slip resistant properties of the trial footwear have been evaluated using the Health and Safety Executive's 'GRIP' rating scheme, which more accurately assesses pedestrian slip potential than the current industry standard tests.
- Results will be generalisable to other industries such as catering, retail, hospitality and manufacturing.
- Slip resistant footwear is one possible measure to help control slip risk for workers, and employers will need to consider whether this is the best solution for their particular working environment.

Introduction

Slips, trips and falls are a common cause of injuries in the workplace. In Great Britain (GB) it is estimated that over 100,000 people are injured due to a slip, trip or fall at work each year, and this represents around 18% of all self-reported non-fatal injuries to workers [1]. The injuries resulting from these incidents can have long-lasting effects. Furthermore, it has been estimated that nearly one million days a year are taken off work due to such injuries [2]. People working in health and social care have one of the highest number of non-fatal slips, trips and falls compared with other industries in GB. It is estimated that there are around 14,000 workers a year injured in this industry sector due to slips, trips and falls (Health and Safety Executive, Labour Force Survey 2018 unpublished data.) This is probably at least partly due to the nature of the flooring on health service premises, which is often very smooth for infection control purposes.

The preference would always be to try to eliminate or adequately control a potential slip risk for all individuals it could affect (workers and members of the public). But this may not always be possible. In this situation, employers may consider the use of slip resistant footwear. Slip risk and the effectiveness of footwear to mitigate it are influenced by many factors such as the slip resistance of the floor surface, the presence and characteristics of any contamination, and the level and type of pedestrian activity. This proposed study will be undertaken in NHS Trusts, which present challenging working environments. They

For peer review only

For peer review only - http://bmjopen.bmj.com/site/about/guidelines.xhtml

have predominantly smooth floor surfaces that become slippery when cleaned or contaminated, there are multiple sources and types of contamination, and there is relatively high pedestrian activity of varied types, e.g. walking, pushing and pulling. Many of the risk factors affecting the healthcare workers participating in the study will be shared by workers in other sectors, such as retail, hospitality, education and manufacturing. Whether it is appropriate to provide slip resistant footwear to control the slip risk can only be determined by means of a risk assessment. The findings of this study will help to inform the risk assessment process and the business case for investing in slip resistant footwear. Many employers already provide footwear to help manage the risk of slips, but a lack of robust testing and reliable information can often lead to inappropriate footwear being selected, and instead of providing a solution, the footwear can add to the problem. This study may help to validate a system by which the slip resistance of footwear can be reliably assessed and gives procurers of footwear the information they need to select footwear with the appropriate level of slip resistance.

There is some evidence that the number of slips occurring in the workplace can be reduced by the use of appropriate slip resistant footwear. An observational study of restaurant workers in the USA, found that the use of slip resistant footwear was associated with a falls reduction of 54% [3]. A before and after study among fisherman suggested slip resistant boots led to a reduction in self-reported slips and falls [4]. However, it can be difficult to specify footwear with appropriate levels of slip resistance. This is because the standard method by which the slip resistance of footwear is assessed, as described in BS EN ISO 13287:2012, does not accurately replicate the action of a slip. Also, the pass criteria do not reflect biomechanical data on the friction requirements for normal walking activities [5]. This has led some to question the validity of this test to predict pedestrian slip potential [6]. Testing footwear under more lifelike conditions allows a more accurate assessment to be made. This has helped to inform the selection of footwear by some companies who have subsequently seen a reduction in accidents and personal liability claims. However, these findings were not in the context of a randomised controlled trial. The SSHeW trial aims to undertake a randomised controlled trial to evaluate the effectiveness and cost effectiveness of NHS Trusts providing slip resistant footwear for its staff, who work in clinical, general and catering environments.

Methods and analysis

Trial design

The SSHeW (Stopping Slips among Healthcare Workers) trial is a pragmatic two-arm, open randomised controlled trial, with an internal pilot, economic evaluation and a qualitative study. In the pilot phase, we will aim to check the feasibility of the study. We will use data from participants recruited during the pilot

For peer review only - http://bmjopen.bmj.com/site/about/guidelines.xhtml

phase to confirm expected recruitment rates, assess attrition and intervention compliance, and calculate the slip rate in the control group. We will readdress the sample size calculation based on these data, and if needed will increase, but not decrease, the target sample size. We aim to recruit 800 participants over the 6-month internal pilot phase. This sample size will allow us to calculate a 90% confidence interval for the slip rate, which would include 7% with a 2% margin of error. Upon successful completion of the internal pilot we will, with the agreement of the funder and the Trial Steering and Data Monitoring and Ethics committee, continue seamlessly with the main trial.

SSHeW study objectives for the pilot phase

1. To test and refine the recruitment strategies for the study.
2. To check the sample size calculation assumptions by reviewing the proportion of participants who experience a slip in the control group.
3. To check the attrition rate.
4. To explore and address any issues regarding footwear compliance.

Prior to the start of recruitment, the funder and the independent Trial Steering Committee (TSC) agreed the following stop/go criteria for progression to the main trial.

1. Recruit at least 400 participants in six months.
2. 80% of the participants will contribute at least 50% of the follow-up text data (i.e. respond to 7/14 weekly post-randomisation text messages).
3. 90% will respond to at least one post-randomisation text.
4. The slip rate in the control group will be at least 7%.

SSHeW main study objective

The primary objective of this research is to assess whether or not the provision of slip resistant footwear to NHS employees working in general, clinical or catering areas will lead to a reduction in the incidence rate of self-reported slips over 14 weeks.

SSHeW secondary objectives

The secondary objectives include:

1. To assess whether or not slip resistant footwear will lead to a reduction in the number of falls (both resulting from a slip or not) over 14 weeks.

For peer review only

For peer review only - http://bmjopen.bmj.com/site/about/guidelines.xhtml

2. To assess whether or not slip resistant footwear will lead to a reduction in the proportion of participants who experience a slip, fall or fracture over 14 weeks.
3. To assess whether or not slip resistant footwear will lead to an increase in the time from randomisation to first slip or fall
4. To assess whether or not the provision of the footwear would be cost-effective.
5. To disseminate the findings of this study using the HSE, NHS Trust managers and Health and Safety managers. This will be in addition to publishing the results of the study in key journals and in a National Institute for Health Research (NIHR) Public Health Research (PHR) report.

Participants

Identification of sites

The study will be undertaken within at least five NHS Trusts in the United Kingdom. A list of the study sites can be obtained from the corresponding author. NHS hospitals are useful organisations for this study as they are large and contain many different working environments. Hospitals have big ecosystem of different 'sub-industries', which will make the trial results generalisable to other industries. For instance, they have large kitchens and staff that work in these are an exemplar of restaurant staff; they have staff working in slippery environments, such as cleaning staff, and these might be similar conditions that exist in food preparation factories or hotels. Hospitals have large numbers of portering staff who need to move heavy loads, similar to supermarket staff. Clinical and ward staff are working on smooth surfaces for infection control purposes, and this will be the case for other healthcare providers in the public and private sector such as in clinics, GP surgeries and nursing homes. Consequently, there are few other institutions that have such a broad range of environments as a hospital. Furthermore, staff turn-over is likely to be relatively lower than in many other commercial organisations, thus minimising the loss to follow-up in our study. Finally, hospital staff are more likely to be used to engaging with research and, therefore, are more likely to take part than staff in other organisations.

Participant recruitment

We will recruit 4,400 NHS staff from a variety of professions, who are subject to a Trust dress code (Figure 1). This will include doctors, nurses, allied health professionals and ward clerks, working in clinical areas e.g. hospital wards, outpatient clinics and service users or patient's homes where clinical activities take place. It will also include catering staff working in catering areas where food is either prepared or served, and porters and cleaners who work throughout clinical, catering and general hospital areas. General areas include all clinical and catering areas in addition to the hospital stairs and corridors. Recruitment will occur for at least 12 months which will capture any potential seasonality of slips.

For peer review only - http://bmjopen.bmj.com/site/about/guidelines.xhtml

Potential participants will be given or sent a recruitment pack of trial information. This may be in response to: 'recruitment days' held within the Trust; postal invitation; posters or flyers located in the Trusts or on the Trusts' intranet or social media pages; or following discussion about the trial at staff meetings. Research and Development (R&D) staff may request a list of NHS employees from their human resources department, and send trial information electronically. The pack will contain an invitation letter, participant information sheet, consent form, baseline questionnaire, list of shoes to choose from and a pre-paid envelope addressed to the York Trials Unit (YTU).

Participants wishing to take part in the SSHeW study will be asked to return their completed consent form and baseline questionnaire to the YTU. Researchers at the YTU will review the responses in the returned baseline questionnaire for participant eligibility for the study according to the inclusion and exclusion criteria. If a person is found to be ineligible for the study, they will be informed in writing, email or by text message. If there are any data queries in the responses to any of the documentation returned by potential participants, then this will be clarified with the participant. Participants may be telephoned, or sent letters, texts or emails. R&D Trust staff may assist with resolving data queries.

All eligible, consenting participants will be sent a copy of their signed consent form and a paper diary to record details of slips, falls and injuries as they occur, and any time off work as a result of an injury caused by a slip or fall. They will be sent a welcome text message followed by four weekly text messages requesting slip data. The wording of the welcome text will be as follows, or similar: "Welcome to the SSHeW trial. We very much value your agreement to participate. You will shortly start to receive text messages asking about any slips you have at work. These texts will always come from this number and will begin with the word SSHeW so that you can recognise them. Thank you."

Eligible participants who respond to at least two of the data collection texts requesting data on slips, irrespective of whether they experience a slip, will be randomised into the trial. Equal randomisation to the intervention group or the waiting list control group will be implemented. The intervention group will be provided with a pair of 5-star GRIP rated slip resistant work shoes by Shoes For Crews®. The control group are expected to wear their usual footwear to work, but will be provided with a pair of 5-star GRIP rated slip resistant shoes by Shoes For Crews® at the end of their participation in the trial. The cost of all shoes, for both groups, is covered by the participating NHS Trusts.

For peer review only - http://bmjopen.bmj.com/site/about/guidelines.xhtml

Please note that this version of the protocol was submitted for publication following the pilot phase of the trial, subsequent to the approval of the following protocol amendment. During the pilot phase, it became apparent that some participants did not respond to their pre-randomisation texts because they had misunderstood and believed that they only had to reply to texts once they received their trial shoes. Approval was sought, and was granted, to send a letter to participants who do not respond to any pre-randomisation texts to reiterate the trial design and processes, and explain that two further text messages will be sent to them. Participants that then go on to respond to both these messages are eligible to be randomised.

Inclusion criteria

Potential participants will be included in the study if they fulfil all of the following criteria:

- Are NHS employees
- Are aged 18 years and over
- Are required to adhere to a dress code policy
- Work at least 60% WTE (22.5 hours per week)
- Have a mobile phone and agree to receive and send outcome data via text messages
- Work in clinical areas (including wards, outpatient clinics, patients' homes), cafeterias, food preparation areas or areas where food is served or in the general hospital environment (including all clinical/catering areas in addition to the hospital stairs and corridors). This will include doctors, nurses, ward clerks, porters and cleaners.

Exclusion criteria

Participants will be ineligible for the SSHeW study if they:

- Are not employed by the NHS
- Do not have a mobile phone or are unwilling/unable to receive/send text messages
- Are provided with footwear by their employer
- Are agency staff, or staff who have less than 6 months remaining on their employment contract
- Work less than 60% WTE (22.5 hours per week) or are predominantly office or theatre based.

Participants who do not wish to take part in the study

Participation in the SSHeW study is voluntary. People who do not wish to take part in the study will not have to return any forms to the YTU.

Randomisation

For peer review only - http://bmjopen.bmj.com/site/about/guidelines.xhtml

Participants who fulfil the eligibility criteria, provide written consent to take part in the study, complete a baseline questionnaire and return at least two weekly texts providing slip data will be eligible for randomisation. Participants will be randomly allocated using the YTU secure web-based randomisation system based on an allocation sequence generated by an independent data systems manager at the YTU , who is not involved in the recruitment of participants. The randomisation will be stratified by NHS Trust, and block randomisation within Trust will be used with variable block sizes. There will be no limit to the size of the block used. This will be dependent on the number of participants to be randomised at the time. Participants will be allocated 1:1 to either the intervention group, to receive a free pair of slip resistant footwear, or the waiting list control group who will be asked to wear their own work footwear for the duration of the study and offered a free pair of slip resistant shoes after completing their follow-up. Participants will be randomised at a particular site in batches, according to when sites state they have capacity to order and deliver footwear. Participants will be notified of their group allocation by a text message, email and/or letter from the YTU. We anticipate that the intervention group will receive their shoes within two weeks of randomisation.

<u>Sample size</u>

There are limited published data on which to base a sample size for this trial. A prospective cohort study [3] found that 49 of 422 workers in a restaurant setting in the USA reported at least one "major" (i.e. resulting in a fall and/or injury) slip over a 12 week follow-up period. We therefore expect that the proportion of workers that experienced any type of slip to be higher than this, though the exact figure is not reported. For our sample size calculation, we require an estimate of the proportion of individuals in the control group that will experience at least one slip over a 14 week follow-up period; we have conservatively assumed a proportion of 10%. We propose to recruit and randomise 4,400 participants using a randomisation ratio of 1:1 (i.e. 2,200 per group). This sample size will give us 90% power to show a 30% relative reduction in the proportion of participants that report at least one slip over a 14 week period (3 percentage point absolute reduction from 10% to 7%) allowing for 20% attrition. It will give us 80% power to see an absolute reduction of 2 percentage points in the risk of falls from 5.5% to 3.5% [3] also allowing for 20% attrition. Although we have based the sample size calculation on detecting a difference in proportions, the primary outcome is the incidence rate of slips over the 14 weeks and so we propose to use a mixed effects Poisson or negative binomial regression model, as appropriate, to compare this outcome between the two groups, which we anticipate will remain adequately powered.

<u>Blinding</u>

For peer review only - http://bmjopen.bmj.com/site/about/guidelines.xhtml

Blinding of participants to group allocation will not be feasible, nor is blinding of the members of the study team who are actively involved in the administration of the study, the statistician or health economist. Data entry staff will be blind to group allocation.

Trial Intervention

SSHeW trial control group

Participants allocated to the control group will be asked to wear their usual work footwear for 14 weeks after they are randomised into the study. At the end of this period they will be offered a free pair of slip resistant shoes provided by 'Shoes for Crews' and paid for by the Trust. It is possible that participants in the control group are already wearing what some would class as slip resistant footwear. The baseline questionnaire will request details of their current footwear style, brand and place of purchase, which will indicate if contamination of the control group has occurred. There is the potential for control group participants to purchase and wear the shoes being evaluated in the trial, as the footwear is commercially available. We believe the likelihood of this happening will be minimised by the fact that control participants will be offered a free pair of trial shoes when their participation in the study has ended. This information will be clearly stated in the study information sheet and control participants will be sent a text eight weeks after randomisation, reminding them that they will receive their new 'Shoes for Crews' footwear at the end of follow-up.

SSHeW trial intervention group

Participants allocated to the intervention group will be offered one pair of 5-star GRIP rated slip resistant footwear provided by 'Shoes for Crews' and paid for by the participating Trust. The footwear has been identified through the use of the HSE GRIP Scheme (www.hsl.gov.uk/products/grip), which measures and categorises the level of wet slip resistance offered by footwear. The 5-star rating is the most effective footwear available. This testing is not part of the normal certification procedure for occupational footwear, but has been shown to be an effective way to differentiate between footwear slip resistance.

Participants will receive one free pair of shoes, which will be selected from a catalogue specifically designed for the trial. In order to assist with the fitting of the footwear, advice provided on the Shoes for Crews website (http://www.sfceurope.com/uk/Footer-Links/About-Us/Shoe-Sizing-Tipsa) will be offered to participants and supported by trial staff where appropriate. The participating NHS Trust will order and pay for the footwear directly.

Outcome measures

For peer review only - http://bmjopen.bmj.com/site/about/guidelines.xhtml

<u>Primary outcome measure for the SSHeW trial</u>

The primary outcome in this study is the incidence rate of self-reported slips, not necessarily resulting in a fall or injury, in the workplace over a 14-week period, as reported via weekly text messages. A slip is defined as 'a loss of traction of your foot on the floor surface, which may or may not result in a fall'. Data will be collected via text messages, sent to/from the participant. Participants will be sent one weekly text message with the following content (or similar):

"SSHeW trial: How many slips did you have at work between DD/MM/20YY and DD/MM/20YY? Please provide a single number (e.g. 2) or 0 if you did not slip. Thank you."

To aid reporting of these events, participants will be given a personal paper weekly diary in which to record if they have a slip or fall, and any resultant injuries. This diary will be sent to them at the start of the study. A fall will be defined as 'an unexpected event in which you come to rest on the ground, floor, or lower level'.

<u>Secondary outcome measures for the SSHeW trial</u>

Secondary outcomes will be self-reported by the participant and collected via text or questionnaires sent at baseline, 14 weeks, or following the report of the first slip or an injury. They include:

- The incidence rate of falls resulting from a slip in the workplace over 14 weeks
- The incidence rate of falls not resulting from a slip in the workplace over 14 weeks
- Proportion of participants who report a slip over 14 weeks
- Proportion of participants who report a fall over 14 weeks
- Proportion of participants who report a fracture over 14 weeks (numbers permitting)
- Time to first slip
- Time to first fall
- EQ5D-5L
- Cost effectiveness

Other important information collected includes:

- Reason for slip/fall, location of fall, type of flooring and if wet or dry, consequence of slip/fall i.e. superficial wound (bruising sprain, cut, abrasions), fractures and type of fractures; severity of fall; type of footwear worn at time of slip/fall
- Number of days off work, due to the slip or fall

For peer review only - http://bmjopen.bmj.com/site/about/guidelines.xhtml

- Footwear worn at time of first slip
- Number of hospital admissions
- Number of days in hospital
- Compliance and reasons for non- compliance
- Any minor injuries resulting from ill-fitting shoes
- Style of footwear usually worn
- Wear on soles of 45 intervention shoes at six, nine and twelve months post randomisation
- Slip resistance of a sample of the current footwear worn by NHS staff

Copies of the data collection forms are available from the corresponding author.

Nested qualitative study

Qualitative interviews will be undertaken in order to determine the acceptability of the footwear. The reasons for wearing or not wearing the footwear and views on the impact of the footwear including unintended consequences will be explored. For instance, there may be certain staff groups for whom it is difficult to store the footwear at work (e.g. absence of personal lockers). We will also interview relevant health service managers, at least one per site, regarding the contextual influences on the use of the footwear. We will purposively select a sample of 30-40 intervention participants, who have completed follow-up, and are a mix of partial and non-adherers (as indicated by their follow-up data) across clinical (e.g. nursing/medical staff) and non-clinical specialties (e.g. cleaning/portering staff) for a brief telephone interview. The maximum variation sampling approach will ensure a collection of a wide range of views [7]. Interviews will be conducted using a topic guide to ensure consistency – although the format will be flexible in order to allow participants to generate naturalistic data on what they see as important.

Adverse events

This study will record and report only details of any serious adverse events (SAEs) that are required to be reported to the Health Research Authority (HRA) i.e. events which are related to taking part in the study and are unexpected. Non-serious adverse events will not be recorded or reported for this study unless they are related to being in the study or are related to the intervention. The Adverse Event reporting period for this trial begins as soon as the participant consents to be in the study and ends approximately 14 weeks after they are randomised i.e. after they are sent their final data collection text message. Adverse events will continue to be collected for participants who agree to long term follow up. For those participants who are not randomised, the reporting period will end once the participant is informed that their participation in the study has ended.

For peer review only - http://bmjopen.bmj.com/site/about/guidelines.xhtml

The most common adverse event likely to occur within this study relates to falls and slips, which are being recorded as an outcome measure of the trial. If a participant has a fall or slip, an adverse event form will not be completed as data are collected elsewhere.

For this trial a Serious Adverse Event (SAE) is defined as any untoward occurrence that:

(a) Results in death
(b) Is life threatening
(c) Requires hospitalisation or prolongation of existing hospitalisation
(d) Consists of a congenital anomaly or birth defect; or
(e) Is otherwise considered medically significant by the investigator

It is expected that some participants may experience minor injuries, resulting from ill-fitting shoes. This may include: blisters, corns, calluses, foot pain, athlete's foot, ingrown toe nails, and general foot pain/discomfort. Occasionally, ill-fitting shoes can cause more persistent foot complaints such as: plantar fasciitis, Morton's neuroma, bursitis or capsulitis which can present as pain, swelling and sometimes numbness in the toes. Structural changes over time can also occur from ill-fitting footwear, for example flat foot or toe deformities such as retracted/hammer/claw/mallet toes and bunions. It is worth noting that the participant may also already have these foot deformities and the shoe style will need to accommodate their altered foot shape. If they are not easily accommodated with the appropriate style of shoe, we can expect that minor injuries will occur and discontinuation of the footwear will be required.

It is expected that some participants will slip, trip or fall during the trial. As a result of such events, participants may require medical treatment, for example treatment of sprains, damage to ligaments, tendons or muscles, or fractures, and may require time off work. In rare cases, participants may require hospitalisation or in extremely rare cases, may be permanently injured or die as a result of a fall or slip. It is also expected that there may be incidents of hospitalisations, illnesses, disabling/ incapacitating/ life-threatening conditions, ageing-associated diseases (such as cancer, cardiovascular disease, diabetes, arthritis, osteoporosis, dementia) and other common illnesses such as depression, and rarely deaths in the study population, such events which are deemed unrelated to the study will not be reported.

An event is defined as 'related' if the event was due to the administration of any research procedure. An 'unexpected event' is defined as a type of event not listed in the protocol as an expected occurrence. The

For peer review only - http://bmjopen.bmj.com/site/about/guidelines.xhtml
For peer review only

relatedness and expectedness of an event will be reviewed by the Chief Investigator and the Trial Steering Committee.

Definition of the end of the trial

The end of the study is defined as the date when the last randomised participant is due to respond to their 14 week follow up text message.

Statistical analysis

There will be two analyses. A descriptive analysis of the internal pilot data and a single effectiveness analysis of the main trial data at end of follow-up of all participants. All analyses will be conducted in STATA v15 or later (StataCorp, 4905 Lakeway Drive, College Station, Texas 77845 USA). Analyses will be described in detail in a Statistical Analysis Plan drafted by the study statisticians and reviewed by the Trial Steering and Data Monitoring and Ethics Committee.

The trial will be reported according to the CONSORT guidelines for clinical trials (Consolidated Standards Of Reporting Trials statement (http://www.consort-statement.org/). Baseline data (sex, age, job role, etc.) will be summarised descriptively overall and by randomised arm, both as randomised and as included in the primary analysis. No formal statistical comparisons of baseline data will be undertaken between the trial arms. Continuous measures will be reported as means and standard deviations whilst categorical data will be reported as counts and percentages. Data will be processed and stored according to the University of York, York Trials Unit's Standard Operating Procedures. Analyses will be conducted following the principles of intention-to-treat with participant's outcomes analysed according to their original, randomised group, where data are available, irrespective of deviations based on non-compliance, unless otherwise stated.

Statistical analysis of the SSHeW primary outcome

Although we have based the sample size calculation on detecting a difference in proportions, the primary outcome is the incidence rate of slips over the 14 weeks of follow-up. We propose to use a mixed effects Poisson or negative binomial regression model (as appropriate depending on the presence of over-dispersion) to compare this outcome between the two groups, which will give us a more powerful analysis. The regression model will adjust for pertinent baseline covariates such as gender, age, job role, and baseline slip rate ascertained from the run-in period. NHS trust will be included as a random effect to account for potential clustering by recruitment site. The number of weeks for which the participant provided weekly slip data and the number of hours worked in those weeks will be accounted for in the

For peer review only - http://bmjopen.bmj.com/site/about/guidelines.xhtml

For peer review only

model. Point estimates in the form of an incident rate ratio and their associated 95% confidence intervals will be provided.

Secondary analysis

All secondary outcomes and other important collected data will be summarised descriptively overall and by trial arm.

The incidence rate of falls (both resulting and not resulting from a slip) over 14 weeks will be analysed in the same way as described above for slips. The following outcomes will be analysed using a mixed effects logistic regression adjusting for the same covariates as the primary analysis and NHS trust as a random effect: (i) the proportion of participants who slip at least once over 14 weeks; (ii) the proportion of participants who fall at least once over 14 weeks; and (iii) subject to a sufficient number of events, the proportion of participants who experience a fracture over 14 weeks. Odds ratios and their associated 95% confidence intervals will be provided.

Time to first slip and the time to first fall will be calculated. Participants who do not report a slip or fall will be treated as censored at their date of trial exit (completion of follow-up or withdrawal). The proportion of participants yet to experience a slip/fall will be summarised by a Kaplan Meier survival curve for each group. Time to slip/fall will be analysed using Cox Proportional Hazards regression, with shared centre frailty and adjusting for the same covariates as in the primary analysis model. Hazard ratios and their associated 95% confidence intervals will be provided. The proportional hazards assumption will be evaluated using Schoenfeld residuals.

Subgroup analyses

We will consider whether the intervention effect differs by gender and area of work by repeating the primary analysis including the factor and an interaction term between the factor and group allocation in the model.

Missing data

The amount of missing data will be reported for each randomised arm, and we will also compare the baseline characteristics of participants who are included in the primary analysis to ensure that any attrition has not produced any imbalance in the groups in important variables. To account for any possible selection bias, a logistic regression will be run to predict non-response (no outcome data received during

For peer review only - http://bmjopen.bmj.com/site/about/guidelines.xhtml

follow-up) including all variables collected prior to randomisation. The primary analysis will then be repeated including as covariates all variables found to be significantly predictive of non-response to determine if this affects the parameter estimates.

Intervention adherence

A Complier Average Causal Effect (CACE) analysis to assess the impact of compliance on treatment estimates will be undertaken. CACE analysis allows an unbiased treatment estimate of, in this case, the incidence rate ratio of slips between the two groups in the presence of non-compliance with the shoes. It is less prone to biased estimates than the more commonly used approaches of per protocol or 'on treatment' analysis as it preserves the original randomisation and uses the randomisation status as an instrumental variable to account for the non-compliance.

Economic evaluation

The health economic evaluation will aim to establish the cost-effectiveness of slip resistant footwear in terms of preventing slips and falls. The economic evaluation will be undertaken in the form of a cost-utility analysis (CUA). It will be conducted from a societal perspective but will also distinguish costs that directly draw on the NHS budget. The trial Health Economist will write a detailed analysis plan prior to any analysis being conducted, which will be reviewed by the TMG and TSC and signed off by the Chief Investigator.

The analysis will estimate total net intervention costs, accounting for (i) implementation costs, primarily footwear purchase costs; and (ii) avoided costs arising from the change in slip rates observed in the trial (reductions in: lost working time due to absenteeism; medical treatment costs; and compensation and legal costs).

We will use data collected during the trial on the consequences of slips, such as type of injury, duration of time off work, and time spent in hospital, and model the effectiveness of the intervention beyond the 14 week time horizon of the trial. With the agreement of the participant, we will collect long term follow up data on the health state (EQ-5D-5L), healthcare resource use, and absence from work, once a month after the 14-week final follow up, from participants reporting an injury. If the participant reports an injury between randomisation but before the 14 week questionnaire then EQ-5D-5L data, information about whether the participants considers they have recovered from the injury and number of days ago they recovered will be collected. Data collection will stop when the injury has resolved, the participant no longer wishes to be contacted or the trial ends. The duration of modelling will depend on the expected lifetime of the footwear. We will gather information on this by asking 15 pilot participants to continue to

For peer review only - http://bmjopen.bmj.com/site/about/guidelines.xhtml

wear their trial shoes for a further six, nine and 12 months (45 participants in total) and then assess the wear of these shoes. This will inform the modelling period used for the economic evaluation.

Table 1 provides an overview of data sources for each of the impacts that will be assessed in the economic evaluation. We will be able to complement this with data from the Health and Safety Executive's Costs to Britain of workplace fatalities and self-reported injuries and ill health ('Costs to Britain') model, which is an established framework used to estimate the economic costs of workplace injuries and ill health for the purposes of annual National Statistics and regulatory impact assessments (http://www.hse.gov.uk/statistics/cost.htm).

Table 1 Data sources for economic evaluation

**Impact**	**Data required**	**Data source**
Intervention costs		
Footwear purchase costs.<br>At the societal level, the purchase of the intervention footwear will displace the purchase or wear of other footwear, so additional costs are likely to be minimal.	Pairs of footwear distributed, unit cost of footwear, effective lifetime of footwear.	Purchase costs already known.<br>Data on effective lifetime of footwear to be collected during follow up of 45 trial participants.
Managerial and staff time incurred in distributing footwear and communicating the intervention	Given that the NHS already has dress requirements and provides staff uniform, we expect that any additional staff time incurred in rolling out the slip resistant footwear will be negligible, so we do not propose to quantify this impact.	
Avoided costs (benefits)		
Loss of productivity/output due to worker absence. Loss of 'production' (in terms of services provided) to the NHS is likely to be minimised where hospitals recruit agency/bank staff to temporarily replace absent workers. The main costs to the NHS from worker absence would therefore be the costs of replacement agency/bank staff.<br><br>At the societal level, a worker unable to work due to injury represents a reduction in the productive capacity of the economy.	Number of full-time equivalent working days lost due to slip-related injuries by type of worker. Average daily costs of agency / bank workers by role (including agency fees)	Trial data on reduction in slip injuries and full-time equivalent days lost, supplemented by the Labour Force Survey data on working days lost due to slips, trips and falls in the healthcare sector.<br><br>Maximum rates for agency wages published by NHS Improvement. Pay rates for bank staff published by NHS Trusts.
Staff sickness payments made to workers absent due to slip-related injury. This is not a cost at the societal level, since the payments	Expected reduction in injuries resulting from slips in the NHS (using data from trial or modelled as discussed later in this protocol), the	Trial data on full time-equivalent days lost as above.<br><br>NHS occupational sick pay

For peer review only - http://bmjopen.bmj.com/site/about/guidelines.xhtml

Impact	Data required	Data source
are a transfer from employer (NHS) to employees.	reduction in time off work associated with these avoided injuries, and NHS occupational sick pay policy (the trial excludes temporary / agency staff).<br><br>Average daily staff costs (wages plus non-wage costs, such as national insurance and pensions contributions).	policy is set out in the NHS Terms of Conditions and Service Handbook. This will be used to model sickness payments based on time off work and staff wage rates.<br><br>NHS staff wage rates by job band publically available. Supplemented by data from the Annual Survey of Hours and Earnings (ASHE) where necessary and the ONS/Eurostat Labour Costs Survey (for non-wage costs).
Healthcare treatment costs incurred due to slip-related injuries	Healthcare resource use, unit healthcare treatment costs.	Data on healthcare resource use to be collected in the study trial (14 –week questionnaire). This will be costed using NHS Reference Costs unit costs database. Supplemented where necessary by published data on healthcare treatment costs for relevant injury types from published sources where available, and HSE 'Costs to Britain' estimates of healthcare treatment costs for injuries.
Compensation (including legal) costs, arising from staff claims following injury under the NHS Liabilities to Third Parties Scheme (LTPS).. At the societal level, the analysis will account for the transfer payment from staff claimants via the scheme.	Average compensation costs to NHS per case due to slip related injuries. This will be based on historical data as any claims from injuries sustained during the trial period are unlikely to be determined before the completion of the study.	NHS Resolution data on non-clinical compensation claims and payments under the NHS Labilities to Third Parties Scheme (LTPS) arising from slip-related staff injuries.
Administrative costs – reporting of slip injuries (RIDDOR), processing sickness payments,<br>dealing with insurance and compensation claims	Amount of staff time spent processing payments, claims etc., plus wage rates of staff.	This is likely to be a small, if not negligible, impact. Could be valued using generic estimates from HSE Costs to Britain model of the typical costs per injury case.

The costing framework applied will ensure that transfers between groups are accounted for (for example, sickness payments), and that costs are not double-counted.

It is anticipated that avoided costs will be driven primarily by avoidance of slips resulting in injury. An estimate from Labour Force Survey data shows that the injury rate from 'slips, trips and falls' in SIC 86

For peer review only - http://bmjopen.bmj.com/site/about/guidelines.xhtml

the 'Human Health Activities' is 0.46% (95% CI 0.38% - 0.55%, averaged 2008/09-2016/17). The rarity of injurious events means that it is likely we will need to model the impact of falls reduction on fall related injuries; data collected from the trial study is likely to be insufficient to enable us to infer a relationship between slips and injuries. Given that the study is unlikely to provide statistically significant results on the change in the injury rate or types of injuries, a central scenario will be to assume that the change in injury rate is commensurate with the observed change in slip rate (i.e. 30% fall in slips results in 30% fall in injuries, and a 30% reduction across all injury types/severities). To facilitate this analysis, we will complement the data collected during the survey with national data on slips reported under the Labour Force Survey, held by HSE under licence from the Office of National Statistics. The LFS provides nationally representative data on self-reported injuries, including the severity of injuries, measured by time off work.

To enable a cost-utility analysis to be undertaken, we will collect EQ-5D-5L data from participants who report an injury and will produce health state profiles, which will be converted to utility scores using published NICE/EuroQol 'standard tariffs'[8]. We will validate this where possible with published studies on the health-related quality of life effects of comparable injuries. We will compare this with age/gender population level data of EQ-5D scores to derive the utility loss associated with slip-related injuries. This will enable the standard cost per QALY measure of cost-effectiveness to be derived. We feel it too onerous and costly to collect EQ-5D from the total trial population as is usual in a trial based economic evaluation, as the vast majority of the participants are healthy and working, will not have an injury, and will return a high utility score. It is proportionate therefore to use existing general population data from published sources.

Two 'threshold' tests will be undertaken:

1. The change in injury rate required to achieve a cost per QALY equal to the NICE threshold of £20,000 to £30,000; and
2. The 'break-even' change in injury rate from the perspective of NHS costs.

The analysis will produce the following results:

- Total net intervention costs (implementation costs minus avoided costs) to the NHS and to society.
- Cost per QALY gained, from both NHS budget and societal perspective.
- Threshold tests, as above.

<u>Qualitative analysis</u>

For peer review only - http://bmjopen.bmj.com/site/about/guidelines.xhtml

All interviews will be audio recorded digitally and transcribed verbatim. A computer package such as ATLAS-ti or NviVo may be used to manage the data. Initially following transcription the data will be analysed using the constant comparison method through thematic coding of the data [9]. Coding will take place using a combination of prior themes and emergent themes. Negative cases will be actively sought throughout the analysis and emerging ideas themes modified in response[10]. Integration of these interview findings with the quantitative data collected in the acceptability questionnaire will be done using a 'triangulation protocol'[11]. This will be done at the data interpretation phase, [12] the data having first been analysed independently. A convergence matrix will be created to display the quantitative and qualitative findings to maintain a sharp focus on the relevance of findings to evaluating the mechanisms of impact for the intervention.

Trial monitoring

The day-to-day management of the study will be monitored by the Trial Management Group. An independent Trial Steering and Data Monitoring Committee will be set up. The committee will meet at least annually or more frequently if the committee requests. Site monitoring will not be undertaken on behalf of the Sponsor since the eligibility for the study is undertaken by review of participant's self-reported data by researchers based at the YTU and consent is taken via the post.

Ethics and dissemination

Ethics

All participants will give written informed consent prior to entry to the study and will be made aware that participation is strictly voluntary. Further consent will be obtained for the qualitative interviews and for testing of footwear for slip resistant properties. Participants may withdraw from the study at any time.

Dissemination

The results of the study will be disseminated through peer-reviewed journals and in the NIHR PHR monograph. The results will also be presented at health and safety conferences, such as The Royal Society for the Prevention of Accidents (ROSPA); Institution of Occupational Safety and Health (IOSH) and The National Examination Board in Occupational Safety and Health (NEBOSH). The Health and Safety Executive will disseminate the findings of the study through their website (www.hse.gov.uk), through direct communications to interested parties such as Health and Safety Managers and via the 'GRIP' scheme. The results will also inform the contents of the 'Slips and Trips - Falls Prevention' training course run by the HSE. A short summary of the results of the study will be produced, which could be distributed to all trial participants and hospital managers at participating trusts.

For peer review only - http://bmjopen.bmj.com/site/about/guidelines.xhtml

Patient and public involvement

Our patient and public representatives were identified from members of the Cheshire and Wirral Partnership's NHS Foundation Trust's Ward Management Task and Finish Group and staff employed by this NHS Trust. NHS employees have helped develop the design and conduct of the study, by providing feedback on the grant application submitted to the funder. They advised on the suitability of the proposed footwear, use and wording of text messages, the method to collect data and the frequency and duration of contacts to ensure an acceptable level of burden and trial documentation. A PPI representative forms part of the trial steering committee to guide the conduct of the study. A summary of the findings will be made available to trial participants.

Studies within trials

In addition to the main SSHeW study, one 'Study within a Trial' (SWAT) is being conducted.

Pen sub-study

The aim of this embedded randomised controlled trial is to evaluate the effectiveness of including a pen with the 14 week follow up questionnaire on response rates to the SSHeW study. Any participant who is due to be sent their 14 week follow up questionnaire will be included in the sub-study. Participants who have withdrawn from follow-up before their 14 week questionnaire is due, or those who have already received their follow-up questionnaire prior to the start of the pen sub-study will be excluded from this sub-study. Simple randomisation in a 1:1 ratio will be used. Generation of the allocation sequence will be undertaken independently by a researcher not involved with sending out the questionnaires. Participants allocated to the pen sub-study intervention group will receive a pen with the University of York/York Trials Unit logo on it with their 14 week questionnaire, whilst pen sub-study control participants will not receive a pen. The primary outcome will be the proportion of participants in each group who return the questionnaire. Secondary outcomes will include time to response (length of time taken to return the questionnaire), completeness of response (the number of questions completed) and whether a reminder notice is required (number of participants requiring a reminder mailing divided by the number of participants who were sent a questionnaire). As is usual with an embedded study within a trial, no formal power calculation will be undertaken for the study, as the sample size will be constrained by the number of participants sent a 14 week questionnaire. This sub-study was introduced during the recruitment period for the main trial when approximately 2,000 participants had already completed their 14 week follow up; only participants due to be followed up after this point were included in the sub-study. Binary data will be

For peer review only - http://bmjopen.bmj.com/site/about/guidelines.xhtml

compared using logistic regression, time to response by a Cox proportional hazards model, and completeness of response by a linear regression model. All models will adjust for main trial allocation.

Discussion

Slips in workers are a major problem. If elimination of the potential slip risk is not possible, then one possible way to reduce the number of slips in the workplace may be to provide slip resistant footwear. Evaluation of slip resistant properties of footwear in slippery conditions is often undertaken using mechanical tests. The standard test method described in BS EN ISO 13287:2012 has been criticised and some have questioned its validity to predict pedestrian slip potential [5, 6]. It has been suggested that testing footwear under more lifelike conditions will more accurately assess the slip resistant properties of the footwear. The Health and Safety Executive have developed the 'GRIP' rating scheme. This scheme measures and categorises the level of wet slip resistance offered by the footwear. Footwear is rated on a scale of one to five stars, with five stars being the highest rating. The footwear used in this study has been assessed and achieved a 5-star rating. One potential limitation to the study is that the study uses unblinded, participant self-report outcome measures, so there is the possibility of reporting bias being introduced. In order to minimise the possibility of resentful demoralisation, the control group will be offered a free pair of trial shoes once their part in the study has ended. The SSHeW protocol aims to evaluate the effectiveness and cost effectiveness of the 5-star rated footwear for the prevention of slips in the workplace. It will be the largest trial of its kind to date. Primary outcome data on the number of slips experienced will be collected weekly via SMS. This is a novel and efficient method of collecting this data. It is quicker, less burdensome and cheaper than participants posting a questionnaire back and, due to the regularity of data collection, minimises the chance of recall bias.

Trial status

Recruitment and follow-up are in progress. The pilot phase of the trial has passed successfully, and the trial has continued seamlessly with no change to the sample size. Recruitment to the study began in March 2016 and will continue until approximately summer 2018. Participants will continue to be followed-up until winter 2018.

Acknowledgments

The research team would like to thank the members of the Trial Steering/Data Monitoring and Ethics Committee: Professor Avril Drummond, Dr Farina Hashmi, Mr Mark Thomas, Kate Chapman and Isabelle Smith for their advice, overseeing the study and reviewing adverse event data. The authors also extend very grateful thanks to the Patient Involvement Group, the study participants and members of the

For peer review only - http://bmjopen.bmj.com/site/about/guidelines.xhtml

For peer review only

Research and Development team for their help with the day-to-day running of the study and to Kevin Hallas from the HSE for his initial work on the study.

Author Contributions

SC and CF wrote the original protocol. DT, SC, CF, GF, CH, ML, MZ HI-S, LG, and RCB were either applicants on the NIHR PHR funding application or helped to refine the protocol. SC wrote the first draft of this article with input from CF and RCB. All authors read and approved the final manuscript.

Funding

This study is funded by the National Institute for Health Research (NIHR) Public Health Research (PHR; Project Reference Number 15/05/28) and the Health and Safety Executive. The views expressed are those of the author(s) and not necessarily those of the NHS, the NIHR, the Department of Health or the Health and Safety Executive. The University of York is the study sponsor and has legal responsibility for the initiation and management of the study: Sponsor representative Mr Michael Barber, Research and Enterprise Directorate, Innovation Office, York Science Park, Heslington, York UK YO10 5DG.

Competing Interest

Catherine Hewitt declares membership of the HTA Commissioning board. The NIHR grant application included some salary costs for SC, CF, GF, CH, ML, MZ, HI-S, LG, RC-B and DT. The HSE grant included some salary costs for ML, GF and MZ. GF and ML declare that the HSE run the GRIP rating scheme, which was used to identify the footwear/manufacture to be used in the trial and Shoes for Crews paid the HSE for testing of footwear under the Grip rating scheme. No other competing interests are declared.

Ethics approval

The study protocol (version 4, 03.08.2017) including the original application and subsequent amendments (as required) received a favourable ethical opinion from the University of York, Department of Health Sciences Research Governance Committee, and, NHS Trust Research and Development at recruiting sites as well as Health Research Authority approval.

Peer review

This study was peer reviewed for ethical and funding approval prior to submission.

Data sharing statement

For peer review only

For peer review only - http://bmjopen.bmj.com/site/about/guidelines.xhtml

Requests to access SSHeW data can be made to the corresponding author once the trial is complete and the trial results published, and will be considered on a case-by-case basis by the Trial Management Group. All data requests will be managed according to York Trials Unit, University of York processes and procedures.

The Office for National Statistics is the provider of the Labour Force Survey data. The analysis of these data presented in this publication is the sole responsibility of the Health and Safety Executive.

REFERENCES

1. Health and Safety Executive. Kind of accident in Great Britain. http://www.hse.gov.uk/statistics/causinj/index.htm2017 [accessed 28.02.2018].
2. Labour Force Survey - Self-reported work-related ill health and workplace injuries: Index of LFS tables. http://www.hse.gov.uk/statistics/lfs/index.htm: Health and Safety Executive 2017.
3. Verma SK, Chang WR, Courtney TK, *al e*. A prospective study of floor surface, shoes, floor cleaning and slipping in US limited-service restaurant workers Occupational Environmental Medicine 2011;**68**:279-85.
4. Jensen OC, Laursen HL. Reduction in slips, trips and falls and better comfort by using new anti-slipping boots in fishing. *International Journal of Injury Control and Safety Promotion* 2010;**1**(18):85-7DOI: 10.1080/17457300.2010.487156.
5. Hallas K, Thorpe S, Liddle M, *et* al. The Role of Safety Footwear in Slipping Accidents. The 17th IEA World Congress on Ergonomics; Beijing,2009.
6. Blanchette MG, Powers CM. Validity and Accuracy of a Slip Resistance Measurement Protocol for the Assessment of Slip Potential. *American Society of Biomechanics* 2011 http://www.asbweb.org/conferences/2011/pdf/38.pdf [accessed 08/02/2013]. .
7. Patton M. Qualitative Evaluation and Research Methods 2nd ed: Newbury Park, CA: Sage; 1990
8. Devlin NJ, Shah KK, Feng Y *et al*. Valuing health-related quality of life: An EQ-5D-5L value set for England. *Health Economics*. 2017;**27**(1):7-22 doi:10.1002/hec.3564.
9. Silverman D. Interpreting Qualitative Data: Methods for Analyzing Talk, Text and Interaction: London: Sage 1993
10. Mays N, Pope C. Rigour and qualitative research. *BMJ* 1995;**311**:109-12.
11. Farmer T, Robinson K, Elliott SJ, *et al*. Developing and Implementing a Triangulation Protocol for Qualitative Health Research. *Qualitataive Health Research* 2006;**16**(3):377-94.
12. O'Cathain A, Murphy E, Nicholl J. Three techniques for integrating data in mixed methods studies. *BMJ*. 2010;341. doi: 10.1136/bmj.c4587.

Figure legend

Figure 1 Flow chart of participants through the pilot phase of the trial and recruitment to the SSHeW main trial.

For peer review only - http://bmjopen.bmj.com/site/about/guidelines.xhtml

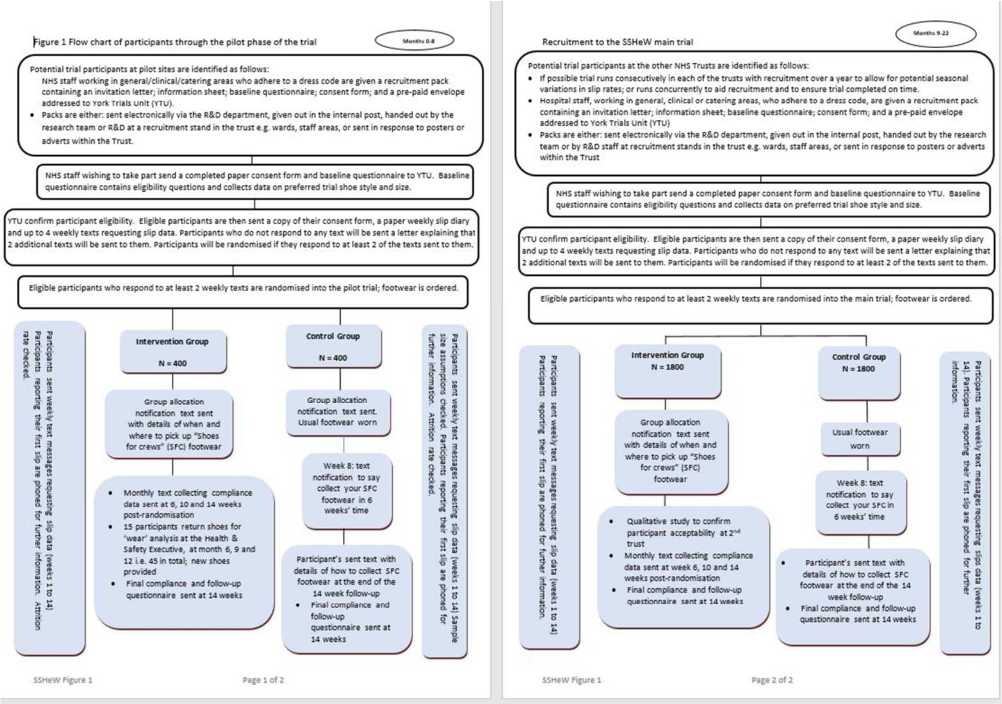

293x206mm (96 x 96 DPI)

For peer review only - http://bmjopen.bmj.com/site/about/guidelines.xhtml

SPIRIT

STANDARD PROTOCOL ITEMS: RECOMMENDATIONS FOR INTERVENTIONAL TRIALS

SPIRIT 2013 Checklist: Recommended items to address in a clinical trial protocol and related documents*

Section/item	Item No	Description	Addressed on page number
**Administrative information**			
Title	1	Descriptive title identifying the study design, population, interventions, and, if applicable, trial acronym	1
Trial registration	2a	Trial identifier and registry name. If not yet registered, name of intended registry	2
	2b	All items from the World Health Organization Trial Registration Data Set	N/A
Protocol version	3	Date and version identifier	24
Funding	4	Sources and types of financial, material, and other support	23
Roles and responsibilities	5a	Names, affiliations, and roles of protocol contributors	1, 23
	5b	Name and contact information for the trial sponsor	1
	5c	Role of study sponsor and funders, if any, in study design; collection, management, analysis, and interpretation of data; writing of the report; and the decision to submit the report for publication, including whether they will have ultimate authority over any of these activities	23
	5d	Composition, roles, and responsibilities of the coordinating centre, steering committee, endpoint adjudication committee, data management team, and other individuals or groups overseeing the trial, if applicable (see Item 21a for data monitoring committee)	23

For peer review only - http://bmjopen.bmj.com/site/about/guidelines.xhtml

**Introduction**			
Background and rationale	6a	Description of research question and justification for undertaking the trial, including summary of relevant studies (published and unpublished) examining benefits and harms for each intervention	3-4
	6b	Explanation for choice of comparators	10,11
Objectives	7	Specific objectives or hypotheses	5
Trial design	8	Description of trial design including type of trial (eg, parallel group, crossover, factorial, single group), allocation ratio, and framework (eg, superiority, equivalence, noninferiority, exploratory)	4, 5
**Methods: Participants, interventions, and outcomes**			
Study setting	9	Description of study settings (eg, community clinic, academic hospital) and list of countries where data will be collected. Reference to where list of study sites can be obtained	3, 4,6,
Eligibility criteria	10	Inclusion and exclusion criteria for participants. If applicable, eligibility criteria for study centres and individuals who will perform the interventions (eg, surgeons, psychotherapists)	8
Interventions	11a	Interventions for each group with sufficient detail to allow replication, including how and when they will be administered	10
	11b	Criteria for discontinuing or modifying allocated interventions for a given trial participant (eg, drug dose change in response to harms, participant request, or improving/worsening disease)	N/A
	11c	Strategies to improve adherence to intervention protocols, and any procedures for monitoring adherence (eg, drug tablet return, laboratory tests)	N/A
	11d	Relevant concomitant care and interventions that are permitted or prohibited during the trial	N/A
Outcomes	12	Primary, secondary, and other outcomes, including the specific measurement variable (eg, systolic blood pressure), analysis metric (eg, change from baseline, final value, time to event), method of aggregation (eg, median, proportion), and time point for each outcome. Explanation of the clinical relevance of chosen efficacy and harm outcomes is strongly recommended	11,12
Participant timeline	13	Time schedule of enrolment, interventions (including any run-ins and washouts), assessments, and visits for participants. A schematic diagram is highly recommended (see Figure)	7, Figure 1,

For peer review only - http://bmjopen.bmj.com/site/about/guidelines.xhtml

Sample size	14	Estimated number of participants needed to achieve study objectives and how it was determined, including clinical and statistical assumptions supporting any sample size calculations	9
Recruitment	15	Strategies for achieving adequate participant enrolment to reach target sample size	7

Methods: Assignment of interventions (for controlled trials)

Allocation:

Sequence generation	16a	Method of generating the allocation sequence (eg, computer-generated random numbers), and list of any factors for stratification. To reduce predictability of a random sequence, details of any planned restriction (eg, blocking) should be provided in a separate document that is unavailable to those who enrol participants or assign interventions	9
Allocation concealment mechanism	16b	Mechanism of implementing the allocation sequence (eg, central telephone; sequentially numbered, opaque, sealed envelopes), describing any steps to conceal the sequence until interventions are assigned	9
Implementation	16c	Who will generate the allocation sequence, who will enrol participants, and who will assign participants to interventions	9
Blinding (masking)	17a	Who will be blinded after assignment to interventions (eg, trial participants, care providers, outcome assessors, data analysts), and how	10
	17b	If blinded, circumstances under which unblinding is permissible, and procedure for revealing a participant's allocated intervention during the trial	N/A

Methods: Data collection, management, and analysis

Data collection methods	18a	Plans for assessment and collection of outcome, baseline, and other trial data, including any related processes to promote data quality (eg, duplicate measurements, training of assessors) and a description of study instruments (eg, questionnaires, laboratory tests) along with their reliability and validity, if known. Reference to where data collection forms can be found, if not in the protocol	11, 12
	18b	Plans to promote participant retention and complete follow-up, including list of any outcome data to be collected for participants who discontinue or deviate from intervention protocols	22

For peer review only - http://bmjopen.bmj.com/site/about/guidelines.xhtml

Data management	19	Plans for data entry, coding, security, and storage, including any related processes to promote data quality (eg, double data entry; range checks for data values). Reference to where details of data management procedures can be found, if not in the protocol	14
Statistical methods	20a	Statistical methods for analysing primary and secondary outcomes. Reference to where other details of the statistical analysis plan can be found, if not in the protocol	15, 16
	20b	Methods for any additional analyses (eg, subgroup and adjusted analyses)	16
	20c	Definition of analysis population relating to protocol non-adherence (eg, as randomised analysis), and any statistical methods to handle missing data (eg, multiple imputation)	16
**Methods: Monitoring**			
Data monitoring	21a	Composition of data monitoring committee (DMC); summary of its role and reporting structure; statement of whether it is independent from the sponsor and competing interests; and reference to where further details about its charter can be found, if not in the protocol. Alternatively, an explanation of why a DMC is not needed	23
	21b	Description of any interim analyses and stopping guidelines, including who will have access to these interim results and make the final decision to terminate the trial	4,5
Harms	22	Plans for collecting, assessing, reporting, and managing solicited and spontaneously reported adverse events and other unintended effects of trial interventions or trial conduct	12 -14
Auditing	23	Frequency and procedures for auditing trial conduct, if any, and whether the process will be independent from investigators and the sponsor	N/A
**Ethics and dissemination**			
Research ethics approval	24	Plans for seeking research ethics committee/institutional review board (REC/IRB) approval	24
Protocol amendments	25	Plans for communicating important protocol modifications (eg, changes to eligibility criteria, outcomes, analyses) to relevant parties (eg, investigators, REC/IRBs, trial participants, trial registries, journals, regulators)	24

For peer review only - http://bmjopen.bmj.com/site/about/guidelines.xhtml

Consent or assent	26a	Who will obtain informed consent or assent from potential trial participants or authorised surrogates, and how (see Item 32)	7, 21
	26b	Additional consent provisions for collection and use of participant data and biological specimens in ancillary studies, if applicable	N/A
Confidentiality	27	How personal information about potential and enrolled participants will be collected, shared, and maintained in order to protect confidentiality before, during, and after the trial	14
Declaration of interests	28	Financial and other competing interests for principal investigators for the overall trial and each study site	24
Access to data	29	Statement of who will have access to the final trial dataset, and disclosure of contractual agreements that limit such access for investigators	24
Ancillary and post-trial care	30	Provisions, if any, for ancillary and post-trial care, and for compensation to those who suffer harm from trial participation	n/a
Dissemination policy	31a	Plans for investigators and sponsor to communicate trial results to participants, healthcare professionals, the public, and other relevant groups (eg, via publication, reporting in results databases, or other data sharing arrangements), including any publication restrictions	1,21
	31b	Authorship eligibility guidelines and any intended use of professional writers	N/A
	31c	Plans, if any, for granting public access to the full protocol, participant-level dataset, and statistical code	24
**Appendices**			
Informed consent materials	32	Model consent form and other related documentation given to participants and authorised surrogates	Available from corresponding author
Biological specimens	33	Plans for collection, laboratory evaluation, and storage of biological specimens for genetic or molecular analysis in the current trial and for future use in ancillary studies, if applicable	N/A

*It is strongly recommended that this checklist be read in conjunction with the SPIRIT 2013 Explanation & Elaboration for important clarification on the items. Amendments to the protocol should be tracked and dated. The SPIRIT checklist is copyrighted by the SPIRIT Group under the Creative Commons "Attribution-NonCommercial-NoDerivs 3.0 Unported" license.

For peer review only - http://bmjopen.bmj.com/site/about/guidelines.xhtml

For peer review only

6

The SSHeW study protocol: Does slip resistant footwear reduce slips among healthcare workers? A randomised controlled trial.

Journal:	*BMJ Open*
Manuscript ID	bmjopen-2018-026023.R1
Article Type:	Protocol
Date Submitted by the Author:	04-Sep-2018
Complete List of Authors:	Cockayne, Sarah; UNIVERSITY OF YORK, Department of Health Sciences<br>Fairhurst, Caroline; University of York, Health Sciences<br>Frost, Gillian; Health and Safety Executive<br>Hewitt, Catherine; The University of York, Health Sciences<br>Liddle, Mark ; Health and Safety Executive , Ergonomics & Human Factors<br>Zand, Michael; Health and Safety Executive<br>Iles-Smith , Heather; Leeds Teaching Hospitals NHS Trust<br>Green , Lorraine; Leeds Biomedical Research Centre<br>Cunningham-Burley, Rachel; University of York, Health Sciences<br>Torgerson, David; The University of York,
**Primary Subject Heading**:	Health services research
Secondary Subject Heading:	Evidence based practice
Keywords:	Slip prevention, slip resistant footwear, randomised controlled trial, short messaging service, national health service

SCHOLARONE™ Manuscripts

The SSHeW study protocol: Does slip resistant footwear reduce slips among healthcare workers? A randomised controlled trial.

Sarah Cockayne[1], Caroline Fairhurst[1], Gillian Frost[2], Catherine Hewitt[1], Mark Liddle[2], Michael Zand[3], Heather Iles-Smith[4], Lorraine Green [4,5], Rachel Cunningham-Burley[1] and David Torgerson[1] on behalf of the SSHeW study

[1] York Trials Unit, Department of Health Sciences, University of York, YO10 5DD, UK

[2] Health and Safety Executive, Buxton, SK17 9JN, UK

[3] Health and Safety Executive, Bootle, L20 7HS, UK

[4] The Leeds Teaching Hospitals NHS Trust, Leeds UK

[5] National Institute for Health Research (NIHR) Leeds Musculoskeletal Biomedical Research Unit, Chapel Allerton Hospital, Leeds, LS7 4SA, UK

$ corresponding author Mrs Sarah Cockayne; University of York, Department of Health Sciences, ARRC Building - Lower Ground Floor, York YO10 5DD. Email sarah.cockayne@york.ac.uk telephone 01904 321736, Fax 01904 321387.

$ SC: sarah.cockayne@york.ac.uk

CF: caroline.fairhurst@york.ac.uk

GF: Gillian.Frost@hse.gov.uk

CH: catherine.hewitt@york.ac.uk

ML: Mark.Liddle@hse.gov.uk

MZ: Michael.Zand@hse.gov.uk

HIS: heather.iles-smith@nhs.net

LG: l.green@leeds.ac.uk

RCB: rachel.cunningham-burley@york.ac.uk

DT: david.torgerson@york.ac.uk

Key words

Slip prevention, slip resistant footwear, randomised controlled trial, National Health Service, short messaging service

Word Count 7,391

For peer review only

For peer review only - http://bmjopen.bmj.com/site/about/guidelines.xhtml

ABSTRACT

Introduction: Slips, trips and falls are common causes of injuries in the workplace. It is estimated that in Great Britain, nearly one million days are taken off work due to these injuries. There is some evidence to suggest this accident burden could be reduced by the use of slip resistant footwear. This protocol describes a multicentre trial investigating the effectiveness and cost-effectiveness of slip resistant footwear to prevent slips in NHS staff working in clinical, general or catering environments.

Methods and analysis: A two-arm, randomised controlled trial conducted within England, with 4,400 NHS staff, aged 18 and above, who adhere to a dress code policy and work in a clinical, catering or general hospital environment. Participants will be randomised 1:1 to the intervention or waiting list control group. The intervention group will be offered a pair of 5-star GRIP rated slip resistant footwear. The control group will be offered the footwear at the end of the trial. The primary outcome is the incidence rate of self-reported slips in the workplace over a 14-week period, as reported via weekly text messages. Secondary outcomes include: time to first slip/fall, proportion of participants who slip and fall over 14 weeks, and incidence rate of falls resulting from and not resulting from a slip in the workplace over 14 weeks. An economic evaluation will assess cost-effectiveness, in terms of cost per quality adjusted life year gained. A nested qualitative study will explore the acceptability of the footwear and compliance.

Ethics and dissemination: This protocol received a favourable ethical opinion from the University of York, Department of Health Sciences Research Governance Committee. The trial results will be published in peer-reviewed journals and at conferences. A summary of the findings will be made available to participants.

Trial registration: Current Controlled Trials ISRCTN33051393, assigned 14/03/2017

For peer review only

For peer review only - http://bmjopen.bmj.com/site/about/guidelines.xhtml

Article Summary

Strengths and limitations of this study

Strengths and limitations of this study

- This is the first randomised controlled trial assessing the effectiveness and cost effectiveness of slip resistant footwear for slip prevention in the UK.
- The slip resistant properties of the trial footwear have been evaluated using the Health and Safety Executive's 'GRIP' rating scheme, which more accurately assesses pedestrian slip potential than the current industry standard tests.
- Results will be generalisable to other industries such as catering, retail, hospitality and manufacturing.
- Slip resistant footwear is one possible measure to help control slip risk for workers, and employers will need to consider whether this is the best solution for their particular working environment.

For peer review only

Introduction

Slips, trips and falls are a common cause of injuries in the workplace. In Great Britain (GB) it is estimated that over 100,000 people are injured due to a slip, trip or fall at work each year, and this represents around 18% of all self-reported non-fatal injuries to workers [1]. The injuries resulting from these incidents can have long-lasting effects. Furthermore, it has been estimated that nearly one million days a year are taken off work due to such injuries [2]. People working in health and social care have one of the highest number of non-fatal slips, trips and falls compared with other industries in GB. It is estimated that there are around 14,000 workers a year injured in this industry sector due to slips, trips and falls (Health and Safety Executive, Labour Force Survey 2018 unpublished data.) This is probably at least partly due to the nature of the flooring on health service premises, which is often very smooth for infection control purposes.

The preference would always be to try to eliminate or adequately control a potential slip risk for all individuals it could affect (workers and members of the public). But this may not always be possible. In this situation, employers may consider the use of slip resistant footwear. Slip risk and the effectiveness of footwear to mitigate it are influenced by many factors such as the slip resistance of the floor surface, the presence and characteristics of any contamination, and the level and type of pedestrian activity. This proposed study will be undertaken in NHS Trusts, which present challenging working environments. They

For peer review only - http://bmjopen.bmj.com/site/about/guidelines.xhtml

have predominantly smooth floor surfaces that become slippery when cleaned or contaminated, there are multiple sources and types of contamination, and there is relatively high pedestrian activity of varied types, e.g. walking, pushing and pulling. Many of the risk factors affecting the healthcare workers participating in the study will be shared by workers in other sectors, such as retail, hospitality, education and manufacturing. Whether it is appropriate to provide slip resistant footwear to control the slip risk can only be determined by means of a risk assessment. The findings of this study will help to inform the risk assessment process and the business case for investing in slip resistant footwear. Many employers already provide footwear to help manage the risk of slips, but a lack of robust testing and reliable information can often lead to inappropriate footwear being selected, and instead of providing a solution, the footwear can add to the problem. This study may help to validate a system by which the slip resistance of footwear can be reliably assessed and gives procurers of footwear the information they need to select footwear with the appropriate level of slip resistance.

There is some evidence that the number of slips occurring in the workplace can be reduced by the use of appropriate slip resistant footwear. An observational study of restaurant workers in the USA, found that the use of slip resistant footwear was associated with a falls reduction of 54% [3]. A before and after study among fisherman suggested slip resistant boots led to a reduction in self-reported slips and falls [4]. However, it can be difficult to specify footwear with appropriate levels of slip resistance. This is because the standard method by which the slip resistance of footwear is assessed, as described in BS EN ISO 13287:2012, does not accurately replicate the action of a slip. Also, the pass criteria do not reflect biomechanical data on the friction requirements for normal walking activities [5]. This has led some to question the validity of this test to predict pedestrian slip potential [6]. Testing footwear under more lifelike conditions allows a more accurate assessment to be made. This has helped to inform the selection of footwear by some companies who have subsequently seen a reduction in accidents and personal liability claims. However, these findings were not in the context of a randomised controlled trial. The SSHeW trial aims to undertake a randomised controlled trial to evaluate the effectiveness and cost effectiveness of NHS Trusts providing slip resistant footwear for its staff, who work in clinical, general and catering environments.

Methods and analysis

Trial design

The SSHeW (Stopping Slips among Healthcare Workers) trial is a pragmatic two-arm, open randomised controlled trial, with an internal pilot, economic evaluation and a qualitative study. In the pilot phase, we will aim to check the feasibility of the study. We will use data from participants recruited during the pilot

For peer review only - http://bmjopen.bmj.com/site/about/guidelines.xhtml

phase to confirm expected recruitment rates, assess attrition and intervention compliance, and calculate the slip rate in the control group. We will readdress the sample size calculation based on these data, and if needed will increase, but not decrease, the target sample size. We aim to recruit 800 participants over the 6-month internal pilot phase. This sample size will allow us to calculate a 90% confidence interval for the slip rate, which would include 7% with a 2% margin of error. Upon successful completion of the internal pilot we will, with the agreement of the funder and the Trial Steering and Data Monitoring and Ethics committee, continue seamlessly with the main trial.

<u>SSHeW study objectives for the pilot phase</u>

1. To test and refine the recruitment strategies for the study.
2. To check the sample size calculation assumptions by reviewing the proportion of participants who experience a slip in the control group.
3. To check the attrition rate.
4. To explore and address any issues regarding footwear compliance.

Prior to the start of recruitment, the funder and the independent Trial Steering Committee (TSC) agreed the following stop/go criteria for progression to the main trial.

1. Recruit at least 400 participants in six months.
2. 80% of the participants will contribute at least 50% of the follow-up text data (i.e. respond to 7/14 weekly post-randomisation text messages).
3. 90% will respond to at least one post-randomisation text.
4. The slip rate in the control group will be at least 7%.

<u>SSHeW main study objective</u>

The primary objective of this research is to assess whether or not the provision of slip resistant footwear to NHS employees working in general, clinical or catering areas will lead to a reduction in the incidence rate of self-reported slips over 14 weeks.

<u>SSHeW secondary objectives</u>

The secondary objectives include:

1. To assess whether or not slip resistant footwear will lead to a reduction in the number of falls (both resulting from a slip or not) over 14 weeks.

For peer review only - http://bmjopen.bmj.com/site/about/guidelines.xhtml
For peer review only

2. To assess whether or not slip resistant footwear will lead to a reduction in the proportion of participants who experience a slip, fall or fracture over 14 weeks.
3. To assess whether or not slip resistant footwear will lead to an increase in the time from randomisation to first slip or fall.
4. To assess whether or not the provision of the footwear would be cost-effective.
5. To disseminate the findings of this study using the HSE, NHS Trust managers and Health and Safety managers. This will be in addition to publishing the results of the study in key journals and in a National Institute for Health Research (NIHR) Public Health Research (PHR) report.

<u>Participants</u>

Identification of sites

The study will be undertaken within at least five NHS Trusts in the United Kingdom. A list of the study sites can be obtained from the corresponding author. NHS hospitals are useful organisations for this study as they are large and contain many different working environments. Hospitals have big ecosystem of different 'sub-industries', which will make the trial results generalisable to other industries. For instance, they have large kitchens and staff that work in these are an exemplar of restaurant staff; they have staff working in slippery environments, such as cleaning staff, and these might be similar conditions that exist in food preparation factories or hotels. Hospitals have large numbers of portering staff who need to move heavy loads, similar to supermarket staff. Clinical and ward staff are working on smooth surfaces for infection control purposes, and this will be the case for other healthcare providers in the public and private sector such as in clinics, GP surgeries and nursing homes. Consequently, there are few other institutions that have such a broad range of environments as a hospital. Furthermore, staff turn-over is likely to be relatively lower than in many other commercial organisations, thus minimising the loss to follow-up in our study. Finally, hospital staff are more likely to be used to engaging with research and, therefore, are more likely to take part than staff in other organisations.

Participant recruitment

We will recruit 4,400 NHS staff from a variety of professions, who are subject to a Trust dress code (Figure 1). This will include doctors, nurses, allied health professionals and ward clerks, working in clinical areas e.g. hospital wards, outpatient clinics and service users or patient's homes where clinical activities take place. It will also include catering staff working in catering areas where food is either prepared or served, and porters and cleaners who work throughout clinical, catering and general hospital areas. General areas include all clinical and catering areas in addition to the hospital stairs and corridors. Recruitment will occur for at least 12 months which will capture any potential seasonality of slips.

For peer review only - http://bmjopen.bmj.com/site/about/guidelines.xhtml

Potential participants will be given or sent a recruitment pack of trial information. This may be in response to: 'recruitment days' held within the Trust; postal invitation; posters or flyers located in the Trusts or on the Trusts' intranet or social media pages; or following discussion about the trial at staff meetings. Research and Development (R&D) staff may request a list of NHS employees from their human resources department, and send trial information electronically. The pack will contain an invitation letter, participant information sheet, consent form, baseline questionnaire, list of shoes to choose from and a pre-paid envelope addressed to the York Trials Unit (YTU).

Participants wishing to take part in the SSHeW study will be asked to return their completed consent form and baseline questionnaire to the YTU. Researchers at the YTU will review the responses in the returned baseline questionnaire for participant eligibility for the study according to the inclusion and exclusion criteria. If a person is found to be ineligible for the study, they will be informed in writing, email or by text message. If there are any data queries in the responses to any of the documentation returned by potential participants, then this will be clarified with the participant. Participants may be telephoned, or sent letters, texts or emails. R&D Trust staff may assist with resolving data queries.

All eligible, consenting participants will be sent a copy of their signed consent form and a paper diary to record details of slips, falls and injuries as they occur, and any time off work as a result of an injury caused by a slip or fall. They will be sent a welcome text message followed by four weekly text messages requesting slip data. The wording of the welcome text will be as follows, or similar: "Welcome to the SSHeW trial. We very much value your agreement to participate. You will shortly start to receive text messages asking about any slips you have at work. These texts will always come from this number and will begin with the word SSHeW so that you can recognise them. Thank you."

Eligible participants who respond to at least two of the data collection texts requesting data on slips, irrespective of whether they experience a slip, will be randomised into the trial. Equal randomisation to the intervention group or the waiting list control group will be implemented. The intervention group will be provided with a pair of 5-star GRIP rated slip resistant work shoes by Shoes For Crews®. The control group are expected to wear their usual footwear to work, but will be provided with a pair of 5-star GRIP rated slip resistant shoes by Shoes For Crews® at the end of their participation in the trial. The cost of all shoes, for both groups, is covered by the participating NHS Trusts.

For peer review only - http://bmjopen.bmj.com/site/about/guidelines.xhtml

Please note that this version of the protocol was submitted for publication following the pilot phase of the trial, subsequent to the approval of the following protocol amendment. During the pilot phase, it became apparent that some participants did not respond to their pre-randomisation texts because they had misunderstood and believed that they only had to reply to texts once they received their trial shoes. Approval was sought, and was granted, to send a letter to participants who do not respond to any pre-randomisation texts to reiterate the trial design and processes, and explain that two further text messages will be sent to them. Participants that then go on to respond to both these messages are eligible to be randomised.

Inclusion criteria

Potential participants will be included in the study if they fulfil all of the following criteria:

- Are NHS employees
- Are aged 18 years and over
- Are required to adhere to a dress code policy
- Work at least 60% WTE (22.5 hours per week)
- Have a mobile phone and agree to receive and send outcome data via text messages
- Work in clinical areas (including wards, outpatient clinics, patients' homes), cafeterias, food preparation areas or areas where food is served or in the general hospital environment (including all clinical/catering areas in addition to the hospital stairs and corridors). This will include doctors, nurses, ward clerks, porters and cleaners.

Exclusion criteria

Participants will be ineligible for the SSHeW study if they:

- Are not employed by the NHS
- Do not have a mobile phone or are unwilling/unable to receive/send text messages
- Are provided with footwear by their employer
- Are agency staff, or staff who have less than 6 months remaining on their employment contract
- Work less than 60% WTE (22.5 hours per week) or are predominantly office or theatre based.

Participants who do not wish to take part in the study

Participation in the SSHeW study is voluntary. People who do not wish to take part in the study will not have to return any forms to the YTU.

Randomisation

For peer review only - http://bmjopen.bmj.com/site/about/guidelines.xhtml
For peer review only

Participants who fulfil the eligibility criteria, provide written consent to take part in the study, complete a baseline questionnaire and return at least two weekly texts providing slip data will be eligible for randomisation. Participants will be randomly allocated using the YTU secure web-based randomisation system based on an allocation sequence generated by an independent data systems manager at the YTU , who is not involved in the recruitment of participants. The randomisation will be stratified by NHS Trust, and block randomisation within Trust will be used with variable block sizes. There will be no limit to the size of the block used. This will be dependent on the number of participants to be randomised at the time. Participants will be allocated 1:1 to either the intervention group, to receive a free pair of slip resistant footwear, or the waiting list control group who will be asked to wear their own work footwear for the duration of the study and offered a free pair of slip resistant shoes after completing their follow-up. Participants will be randomised at a particular site in batches, according to when sites state they have capacity to order and deliver footwear. Participants will be notified of their group allocation by a text message, email and/or letter from the YTU. We anticipate that the intervention group will receive their shoes within two weeks of randomisation.

<u>Sample size</u>

There are limited published data on which to base a sample size for this trial. A prospective cohort study [3] found that 49 of 422 workers in a restaurant setting in the USA reported at least one “major” (i.e. resulting in a fall and/or injury) slip over a 12 week follow-up period. We therefore expect that the proportion of workers that experienced any type of slip to be higher than this, though the exact figure is not reported. For our sample size calculation, we require an estimate of the proportion of individuals in the control group that will experience at least one slip over a 14 week follow-up period; we have conservatively assumed a proportion of 10%. We propose to recruit and randomise 4,400 participants using a randomisation ratio of 1:1 (i.e. 2,200 per group). This sample size will give us 90% power to show a 30% relative reduction in the proportion of participants that report at least one slip over a 14 week period (3 percentage point absolute reduction from 10% to 7%) allowing for 20% attrition. It will give us 80% power to see an absolute reduction of 2 percentage points in the risk of falls from 5.5% to 3.5% [3] also allowing for 20% attrition. Although we have based the sample size calculation on detecting a difference in proportions, the primary outcome is the incidence rate of slips over the 14 weeks and so we propose to use a mixed effects Poisson or negative binomial regression model, as appropriate, to compare this outcome between the two groups, which we anticipate will remain adequately powered.

<u>Blinding</u>

For peer review only - http://bmjopen.bmj.com/site/about/guidelines.xhtml

Blinding of participants to group allocation will not be feasible, nor is blinding of the members of the study team who are actively involved in the administration of the study, the statistician or health economist. Data entry staff will be blind to group allocation.

Trial Intervention

SSHeW trial control group

Participants allocated to the control group will be asked to wear their usual work footwear for 14 weeks after they are randomised into the study. At the end of this period they will be offered a free pair of slip resistant shoes provided by 'Shoes for Crews' and paid for by the Trust. It is possible that participants in the control group are already wearing what some would class as slip resistant footwear. The baseline questionnaire will request details of their current footwear style, brand and place of purchase, which will indicate if contamination of the control group has occurred. There is the potential for control group participants to purchase and wear the shoes being evaluated in the trial, as the footwear is commercially available. We believe the likelihood of this happening will be minimised by the fact that control participants will be offered a free pair of trial shoes when their participation in the study has ended. This information will be clearly stated in the study information sheet and control participants will be sent a text eight weeks after randomisation, reminding them that they will receive their new ‘Shoes for Crews’ footwear at the end of follow-up.

SSHeW trial intervention group

Participants allocated to the intervention group will be offered one pair of 5-star GRIP rated slip resistant footwear provided by ‘Shoes for Crews’ and paid for by the participating Trust. The footwear has been identified through the use of the HSE GRIP Scheme (www.hsl.gov.uk/products/grip), which measures and categorises the level of wet slip resistance offered by footwear. The 5-star rating is the most effective footwear available. This testing is not part of the normal certification procedure for occupational footwear, but has been shown to be an effective way to differentiate between footwear slip resistance.

Participants will receive one free pair of shoes, which will be selected from a catalogue specifically designed for the trial. In order to assist with the fitting of the footwear, advice provided on the Shoes for Crews website (http://www.sfceurope.com/uk/Footer-Links/About-Us/Shoe-Sizing-Tipsa) will be offered to participants and supported by trial staff where appropriate. The participating NHS Trust will order and pay for the footwear directly.

Outcome measures

For peer review only - http://bmjopen.bmj.com/site/about/guidelines.xhtml

Primary outcome measure for the SSHeW trial

The primary outcome in this study is the incidence rate of self-reported slips, not necessarily resulting in a fall or injury, in the workplace over a 14-week period, as reported via weekly text messages. A slip is defined as 'a loss of traction of your foot on the floor surface, which may or may not result in a fall'. Data will be collected via text messages, sent to/from the participant. Participants will be sent one weekly text message with the following content (or similar):

"SSHeW trial: How many slips did you have at work between DD/MM/20YY and DD/MM/20YY? Please provide a single number (e.g. 2) or 0 if you did not slip. Thank you."

To aid reporting of these events, participants will be given a personal paper weekly diary in which to record if they have a slip or fall, and any resultant injuries. This diary will be sent to them at the start of the study. A fall will be defined as 'an unexpected event in which you come to rest on the ground, floor, or lower level'.

Secondary outcome measures for the SSHeW trial

Secondary outcomes will be self-reported by the participant and collected via text or questionnaires sent at baseline, 14 weeks, or following the report of the first slip or an injury. They include:

- The incidence rate of falls resulting from a slip in the workplace over 14 weeks
- The incidence rate of falls not resulting from a slip in the workplace over 14 weeks
- Proportion of participants who report a slip over 14 weeks
- Proportion of participants who report a fall over 14 weeks
- Proportion of participants who report a fracture over 14 weeks (numbers permitting)
- Time to first slip
- Time to first fall
- EQ5D-5L
- Cost effectiveness

Other important information collected includes:

- Reason for slip/fall, location of fall, type of flooring and if wet or dry, consequence of slip/fall i.e. superficial wound (bruising sprain, cut, abrasions), fractures and type of fractures; severity of fall; type of footwear worn at time of slip/fall
- Number of days off work, due to the slip or fall

For peer review only - http://bmjopen.bmj.com/site/about/guidelines.xhtml

- Footwear worn at time of first slip
- Number of hospital admissions
- Number of days in hospital
- Compliance and reasons for non- compliance
- Any minor injuries resulting from ill-fitting shoes
- Style of footwear usually worn
- Wear on soles of 45 intervention shoes at six, nine and twelve months post randomisation
- Slip resistance of a sample of the current footwear worn by NHS staff

Copies of the data collection forms are available from the corresponding author.

<u>Nested qualitative study</u>

Qualitative interviews will be undertaken in order to determine the acceptability of the footwear. The reasons for wearing or not wearing the footwear and views on the impact of the footwear including unintended consequences will be explored. For instance, there may be certain staff groups for whom it is difficult to store the footwear at work (e.g. absence of personal lockers). We will also interview relevant health service managers, at least one per site, regarding the contextual influences on the use of the footwear. We will purposively select a sample of 30-40 intervention participants, who have completed follow-up, and are a mix of partial and non-adherers (as indicated by their follow-up data) across clinical (e.g. nursing/medical staff) and non-clinical specialties (e.g. cleaning/portering staff) for a brief telephone interview. The maximum variation sampling approach will ensure a collection of a wide range of views [7]. Interviews will be conducted using a topic guide to ensure consistency – although the format will be flexible in order to allow participants to generate naturalistic data on what they see as important.

<u>Adverse events</u>

This study will record and report only details of any serious adverse events (SAEs) that are required to be reported to the Health Research Authority (HRA) i.e. events which are related to taking part in the study and are unexpected. Non-serious adverse events will not be recorded or reported for this study unless they are related to being in the study or are related to the intervention. The Adverse Event reporting period for this trial begins as soon as the participant consents to be in the study and ends approximately 14 weeks after they are randomised i.e. after they are sent their final data collection text message. Adverse events will continue to be collected for participants who agree to long term follow up. For those participants who are not randomised, the reporting period will end once the participant is informed that their participation in the study has ended.

For peer review only - http://bmjopen.bmj.com/site/about/guidelines.xhtml

The most common adverse event likely to occur within this study relates to falls and slips, which are being recorded as an outcome measure of the trial. If a participant has a fall or slip, an adverse event form will not be completed as data are collected elsewhere.

For this trial a Serious Adverse Event (SAE) is defined as any untoward occurrence that:

(a) Results in death
(b) Is life threatening
(c) Requires hospitalisation or prolongation of existing hospitalisation
(d) Consists of a congenital anomaly or birth defect; or
(e) Is otherwise considered medically significant by the investigator

It is expected that some participants may experience minor injuries, resulting from ill-fitting shoes. This may include: blisters, corns, calluses, foot pain, athlete's foot, ingrown toe nails, and general foot pain/discomfort. Occasionally, ill-fitting shoes can cause more persistent foot complaints such as: plantar fasciitis, Morton's neuroma, bursitis or capsulitis which can present as pain, swelling and sometimes numbness in the toes. Structural changes over time can also occur from ill-fitting footwear, for example flat foot or toe deformities such as retracted/hammer/claw/mallet toes and bunions. It is worth noting that the participant may also already have these foot deformities and the shoe style will need to accommodate their altered foot shape. If they are not easily accommodated with the appropriate style of shoe, we can expect that minor injuries will occur and discontinuation of the footwear will be required.

It is expected that some participants will slip, trip or fall during the trial. As a result of such events, participants may require medical treatment, for example treatment of sprains, damage to ligaments, tendons or muscles, or fractures, and may require time off work. In rare cases, participants may require hospitalisation or in extremely rare cases, may be permanently injured or die as a result of a fall or slip. It is also expected that there may be incidents of hospitalisations, illnesses, disabling/ incapacitating/ life-threatening conditions, ageing-associated diseases (such as cancer, cardiovascular disease, diabetes, arthritis, osteoporosis, dementia) and other common illnesses such as depression, and rarely deaths in the study population, such events which are deemed unrelated to the study will not be reported.

An event is defined as 'related' if the event was due to the administration of any research procedure. An 'unexpected event' is defined as a type of event not listed in the protocol as an expected occurrence. The

For peer review only - http://bmjopen.bmj.com/site/about/guidelines.xhtml

relatedness and expectedness of an event will be reviewed by the Chief Investigator and the Trial Steering Committee.

Definition of the end of the trial

The end of the study is defined as the date when the last randomised participant is due to respond to their 14 week follow up text message.

Statistical analysis

There will be two analyses. A descriptive analysis of the internal pilot data and a single effectiveness analysis of the main trial data at end of follow-up of all participants. All analyses will be conducted in STATA v15 or later (StataCorp, 4905 Lakeway Drive, College Station, Texas 77845 USA). Analyses will be described in detail in a Statistical Analysis Plan drafted by the study statisticians and reviewed by the Trial Steering and Data Monitoring and Ethics Committee.

The trial will be reported according to the CONSORT guidelines for clinical trials (Consolidated Standards Of Reporting Trials statement (http://www.consort-statement.org/). Baseline data (sex, age, job role, etc.) will be summarised descriptively overall and by randomised arm, both as randomised and as included in the primary analysis. No formal statistical comparisons of baseline data will be undertaken between the trial arms. Continuous measures will be reported as means and standard deviations whilst categorical data will be reported as counts and percentages. Data will be processed and stored according to the University of York, York Trials Unit's Standard Operating Procedures. Analyses will be conducted following the principles of intention-to-treat with participant's outcomes analysed according to their original, randomised group, where data are available, irrespective of deviations based on non-compliance, unless otherwise stated.

Statistical analysis of the SSHeW primary outcome

Although we have based the sample size calculation on detecting a difference in proportions, the primary outcome is the incidence rate of slips over the 14 weeks of follow-up. We propose to use a mixed effects Poisson or negative binomial regression model (as appropriate depending on the presence of over-dispersion) to compare this outcome between the two groups, which will give us a more powerful analysis. The regression model will adjust for pertinent baseline covariates such as gender, age, job role, and baseline slip rate ascertained from the run-in period. NHS trust will be included as a random effect to account for potential clustering by recruitment site. The number of weeks for which the participant provided weekly slip data and the number of hours worked in those weeks will be accounted for in the

For peer review only - http://bmjopen.bmj.com/site/about/guidelines.xhtml

model. Point estimates in the form of an incident rate ratio and their associated 95% confidence intervals will be provided.

Secondary analysis

All secondary outcomes and other important collected data will be summarised descriptively overall and by trial arm.

The incidence rate of falls (both resulting and not resulting from a slip) over 14 weeks will be analysed in the same way as described above for slips. The following outcomes will be analysed using a mixed effects logistic regression adjusting for the same covariates as the primary analysis and NHS trust as a random effect: (i) the proportion of participants who slip at least once over 14 weeks; (ii) the proportion of participants who fall at least once over 14 weeks; and (iii) subject to a sufficient number of events, the proportion of participants who experience a fracture over 14 weeks. Odds ratios and their associated 95% confidence intervals will be provided.

Time to first slip and the time to first fall will be calculated. Participants who do not report a slip or fall will be treated as censored at their date of trial exit (completion of follow-up or withdrawal). The proportion of participants yet to experience a slip/fall will be summarised by a Kaplan Meier survival curve for each group. Time to slip/fall will be analysed using Cox Proportional Hazards regression, with shared centre frailty and adjusting for the same covariates as in the primary analysis model. Hazard ratios and their associated 95% confidence intervals will be provided. The proportional hazards assumption will be evaluated using Schoenfeld residuals.

Subgroup analyses

We will consider whether the intervention effect differs by gender and area of work by repeating the primary analysis including the factor and an interaction term between the factor and group allocation in the model.

Missing data

The amount of missing data will be reported for each randomised arm, and we will also compare the baseline characteristics of participants who are included in the primary analysis to ensure that any attrition has not produced any imbalance in the groups in important variables. To account for any possible selection bias, a logistic regression will be run to predict non-response (no outcome data received during

For peer review only - http://bmjopen.bmj.com/site/about/guidelines.xhtml
For peer review only

follow-up) including all variables collected prior to randomisation. The primary analysis will then be repeated including as covariates all variables found to be significantly predictive of non-response to determine if this affects the parameter estimates.

Intervention adherence

A Complier Average Causal Effect (CACE) analysis to assess the impact of compliance on treatment estimates will be undertaken. CACE analysis allows an unbiased treatment estimate of, in this case, the incidence rate ratio of slips between the two groups in the presence of non-compliance with the shoes. It is less prone to biased estimates than the more commonly used approaches of per protocol or 'on treatment' analysis as it preserves the original randomisation and uses the randomisation status as an instrumental variable to account for the non-compliance.

Economic evaluation

The health economic evaluation will aim to establish the cost-effectiveness of slip resistant footwear in terms of preventing slips and falls. The economic evaluation will be undertaken in the form of a cost-utility analysis (CUA). It will be conducted from a societal perspective but will also distinguish costs that directly draw on the NHS budget. The trial Health Economist will write a detailed analysis plan prior to any analysis being conducted, which will be reviewed by the TMG and TSC and signed off by the Chief Investigator.

The analysis will estimate total net intervention costs, accounting for (i) implementation costs, primarily footwear purchase costs; and (ii) avoided costs arising from the change in slip rates observed in the trial (reductions in: lost working time due to absenteeism; medical treatment costs; and compensation and legal costs).

We will use data collected during the trial on the consequences of slips, such as type of injury, duration of time off work, and time spent in hospital, and model the effectiveness of the intervention beyond the 14 week time horizon of the trial. With the agreement of the participant, we will collect long term follow up data on the health state (EQ-5D-5L), healthcare resource use, and absence from work, once a month after the 14-week final follow up, from participants reporting an injury. If the participant reports an injury between randomisation but before the 14 week questionnaire then EQ-5D-5L data, information about whether the participants considers they have recovered from the injury and number of days ago they recovered will be collected. Data collection will stop when the injury has resolved, the participant no longer wishes to be contacted or the trial ends. The duration of modelling will depend on the expected lifetime of the footwear. We will gather information on this by asking 15 pilot participants to continue to

For peer review only - http://bmjopen.bmj.com/site/about/guidelines.xhtml

wear their trial shoes for a further six, nine and 12 months (45 participants in total) and then assess the wear of these shoes. This will inform the modelling period used for the economic evaluation.

Table 1 provides an overview of data sources for each of the impacts that will be assessed in the economic evaluation. We will be able to complement this with data from the Health and Safety Executive's Costs to Britain of workplace fatalities and self-reported injuries and ill health ('Costs to Britain') model, which is an established framework used to estimate the economic costs of workplace injuries and ill health for the purposes of annual National Statistics and regulatory impact assessments (http://www.hse.gov.uk/statistics/cost.htm).

Table 1 Data sources for economic evaluation

**Impact**	**Data required**	**Data source**
Intervention costs		
Footwear purchase costs.<br>At the societal level, the purchase of the intervention footwear will displace the purchase or wear of other footwear, so additional costs are likely to be minimal.	Pairs of footwear distributed, unit cost of footwear, effective lifetime of footwear.	Purchase costs already known. Data on effective lifetime of footwear to be collected during follow up of 45 trial participants.
Managerial and staff time incurred in distributing footwear and communicating the intervention	Given that the NHS already has dress requirements and provides staff uniform, we expect that any additional staff time incurred in rolling out the slip resistant footwear will be negligible, so we do not propose to quantify this impact.	
Avoided costs (benefits)		
Loss of productivity/output due to worker absence. Loss of 'production' (in terms of services provided) to the NHS is likely to be minimised where hospitals recruit agency/bank staff to temporarily replace absent workers. The main costs to the NHS from worker absence would therefore be the costs of replacement agency/bank staff.<br><br>At the societal level, a worker unable to work due to injury represents a reduction in the productive capacity of the economy.	Number of full-time equivalent working days lost due to slip-related injuries by type of worker. Average daily costs of agency / bank workers by role (including agency fees)	Trial data on reduction in slip injuries and full-time equivalent days lost, supplemented by the Labour Force Survey data on working days lost due to slips, trips and falls in the healthcare sector.<br><br>Maximum rates for agency wages published by NHS Improvement. Pay rates for bank staff published by NHS Trusts.
Staff sickness payments made to workers absent due to slip-related injury. This is not a cost at the societal level, since the payments	Expected reduction in injuries resulting from slips in the NHS (using data from trial or modelled as discussed later in this protocol), the	Trial data on full time-equivalent days lost as above.<br><br>NHS occupational sick pay

For peer review only - http://bmjopen.bmj.com/site/about/guidelines.xhtml

Impact	Data required	Data source
are a transfer from employer (NHS) to employees.	reduction in time off work associated with these avoided injuries, and NHS occupational sick pay policy (the trial excludes temporary / agency staff).<br><br>Average daily staff costs (wages plus non-wage costs, such as national insurance and pensions contributions).	policy is set out in the NHS Terms of Conditions and Service Handbook. This will be used to model sickness payments based on time off work and staff wage rates.<br><br>NHS staff wage rates by job band publically available. Supplemented by data from the Annual Survey of Hours and Earnings (ASHE) where necessary and the ONS/Eurostat Labour Costs Survey (for non-wage costs).
Healthcare treatment costs incurred due to slip-related injuries	Healthcare resource use, unit healthcare treatment costs.	Data on healthcare resource use to be collected in the study trial (14 –week questionnaire). This will be costed using NHS Reference Costs unit costs database. Supplemented where necessary by published data on healthcare treatment costs for relevant injury types from published sources where available, and HSE 'Costs to Britain' estimates of healthcare treatment costs for injuries.
Compensation (including legal) costs, arising from staff claims following injury under the NHS Liabilities to Third Parties Scheme (LTPS).. At the societal level, the analysis will account for the transfer payment from staff claimants via the scheme.	Average compensation costs to NHS per case due to slip related injuries. This will be based on historical data as any claims from injuries sustained during the trial period are unlikely to be determined before the completion of the study.	NHS Resolution data on non-clinical compensation claims and payments under the NHS Labilities to Third Parties Scheme (LTPS) arising from slip-related staff injuries.
Administrative costs – reporting of slip injuries (RIDDOR), processing sickness payments,<br>dealing with insurance and compensation claims	Amount of staff time spent processing payments, claims etc., plus wage rates of staff.	This is likely to be a small, if not negligible, impact. Could be valued using generic estimates from HSE Costs to Britain model of the typical costs per injury case.

The costing framework applied will ensure that transfers between groups are accounted for (for example, sickness payments), and that costs are not double-counted.

It is anticipated that avoided costs will be driven primarily by avoidance of slips resulting in injury. An estimate from Labour Force Survey data shows that the injury rate from 'slips, trips and falls' in SIC 86

For peer review only - http://bmjopen.bmj.com/site/about/guidelines.xhtml
For peer review only

the ‘Human Health Activities’ is 0.46% (95% CI 0.38% - 0.55%, averaged 2008/09-2016/17). The rarity of injurious events means that it is likely we will need to model the impact of falls reduction on fall related injuries; data collected from the trial study is likely to be insufficient to enable us to infer a relationship between slips and injuries. Given that the study is unlikely to provide statistically significant results on the change in the injury rate or types of injuries, a central scenario will be to assume that the change in injury rate is commensurate with the observed change in slip rate (i.e. 30% fall in slips results in 30% fall in injuries, and a 30% reduction across all injury types/severities). To facilitate this analysis, we will complement the data collected during the survey with national data on slips reported under the Labour Force Survey, held by HSE under licence from the Office of National Statistics. The LFS provides nationally representative data on self-reported injuries, including the severity of injuries, measured by time off work.

To enable a cost-utility analysis to be undertaken, we will collect EQ-5D-5L data from participants who report an injury and will produce health state profiles, which will be converted to utility scores using published NICE/EuroQol ‘standard tariffs’[8]. We will validate this where possible with published studies on the health-related quality of life effects of comparable injuries. We will compare this with age/gender population level data of EQ-5D scores to derive the utility loss associated with slip-related injuries. This will enable the standard cost per QALY measure of cost-effectiveness to be derived. We feel it too onerous and costly to collect EQ-5D from the total trial population as is usual in a trial based economic evaluation, as the vast majority of the participants are healthy and working, will not have an injury, and will return a high utility score. It is proportionate therefore to use existing general population data from published sources.

Two ‘threshold’ tests will be undertaken:

1. The change in injury rate required to achieve a cost per QALY equal to the NICE threshold of £20,000 to £30,000; and
2. The ‘break-even’ change in injury rate from the perspective of NHS costs.

The analysis will produce the following results:

- Total net intervention costs (implementation costs minus avoided costs) to the NHS and to society.
- Cost per QALY gained, from both NHS budget and societal perspective.
- Threshold tests, as above.

<u>Qualitative analysis</u>

For peer review only - http://bmjopen.bmj.com/site/about/guidelines.xhtml

All interviews will be audio recorded digitally and transcribed verbatim. A computer package such as ATLAS-ti or NviVo may be used to manage the data. Initially following transcription the data will be analysed using the constant comparison method through thematic coding of the data [9]. Coding will take place using a combination of prior themes and emergent themes. Negative cases will be actively sought throughout the analysis and emerging ideas themes modified in response[10]. Integration of these interview findings with the quantitative data collected in the acceptability questionnaire will be done using a 'triangulation protocol'[11]. This will be done at the data interpretation phase, [12] the data having first been analysed independently. A convergence matrix will be created to display the quantitative and qualitative findings to maintain a sharp focus on the relevance of findings to evaluating the mechanisms of impact for the intervention.

For peer review only

Trial monitoring

The day-to-day management of the study will be monitored by the Trial Management Group. An independent Trial Steering and Data Monitoring Committee will be set up. The committee will meet at least annually or more frequently if the committee requests. Site monitoring will not be undertaken on behalf of the Sponsor since the eligibility for the study is undertaken by review of participant's self-reported data by researchers based at the YTU and consent is taken via the post.

Ethics and dissemination

Ethics

All participants will give written informed consent prior to entry to the study and will be made aware that participation is strictly voluntary. Further consent will be obtained for the qualitative interviews and for testing of footwear for slip resistant properties. Participants may withdraw from the study at any time.

Dissemination

The results of the study will be disseminated through peer-reviewed journals and in the NIHR PHR monograph. The results will also be presented at health and safety conferences, such as The Royal Society for the Prevention of Accidents (ROSPA); Institution of Occupational Safety and Health (IOSH) and The National Examination Board in Occupational Safety and Health (NEBOSH). The Health and Safety Executive will disseminate the findings of the study through their website (www.hse.gov.uk), through direct communications to interested parties such as Health and Safety Managers and via the 'GRIP' scheme. The results will also inform the contents of the 'Slips and Trips - Falls Prevention' training course run by the HSE. A short summary of the results of the study will be produced, which could be distributed to all trial participants and hospital managers at participating trusts.

For peer review only - http://bmjopen.bmj.com/site/about/guidelines.xhtml

Patient and public involvement

Our patient and public representatives were identified from members of the Cheshire and Wirral Partnership's NHS Foundation Trust's Ward Management Task and Finish Group and staff employed by this NHS Trust. NHS employees have helped develop the design and conduct of the study, by providing feedback on the grant application submitted to the funder. They advised on the suitability of the proposed footwear, use and wording of text messages, the method to collect data and the frequency and duration of contacts to ensure an acceptable level of burden and trial documentation. A PPI representative forms part of the trial steering committee to guide the conduct of the study. A summary of the findings will be made available to trial participants.

Studies within trials

In addition to the main SSHeW study, one 'Study within a Trial' (SWAT) is being conducted.

Pen sub-study

The aim of this embedded randomised controlled trial is to evaluate the effectiveness of including a pen with the 14 week follow up questionnaire on response rates to the SSHeW study. Any participant who is due to be sent their 14 week follow up questionnaire will be included in the sub-study. Participants who have withdrawn from follow-up before their 14 week questionnaire is due, or those who have already received their follow-up questionnaire prior to the start of the pen sub-study will be excluded from this sub-study. Simple randomisation in a 1:1 ratio will be used. Generation of the allocation sequence will be undertaken independently by a researcher not involved with sending out the questionnaires. Participants allocated to the pen sub-study intervention group will receive a pen with the University of York/York Trials Unit logo on it with their 14 week questionnaire, whilst pen sub-study control participants will not receive a pen. The primary outcome will be the proportion of participants in each group who return the questionnaire. Secondary outcomes will include time to response (length of time taken to return the questionnaire), completeness of response (the number of questions completed) and whether a reminder notice is required (number of participants requiring a reminder mailing divided by the number of participants who were sent a questionnaire). As is usual with an embedded study within a trial, no formal power calculation will be undertaken for the study, as the sample size will be constrained by the number of participants sent a 14 week questionnaire. This sub-study was introduced during the recruitment period for the main trial when approximately 2,000 participants had already completed their 14 week follow up; only participants due to be followed up after this point were included in the sub-study. Binary data will be

For peer review only - http://bmjopen.bmj.com/site/about/guidelines.xhtml

compared using logistic regression, time to response by a Cox proportional hazards model, and completeness of response by a linear regression model. All models will adjust for main trial allocation.

Discussion

Slips in workers are a major problem. If elimination of the potential slip risk is not possible, then one possible way to reduce the number of slips in the workplace may be to provide slip resistant footwear. Evaluation of slip resistant properties of footwear in slippery conditions is often undertaken using mechanical tests. The standard test method described in BS EN ISO 13287:2012 has been criticised and some have questioned its validity to predict pedestrian slip potential [5, 6]. It has been suggested that testing footwear under more lifelike conditions will more accurately assess the slip resistant properties of the footwear. The Health and Safety Executive have developed the 'GRIP' rating scheme. This scheme measures and categorises the level of wet slip resistance offered by the footwear. Footwear is rated on a scale of one to five stars, with five stars being the highest rating. The footwear used in this study has been assessed and achieved a 5-star rating. One potential limitation to the study is that the study uses unblinded, participant self-report outcome measures, so there is the possibility of reporting bias being introduced. In order to minimise the possibility of resentful demoralisation, the control group will be offered a free pair of trial shoes once their part in the study has ended. The SSHeW protocol aims to evaluate the effectiveness and cost effectiveness of the 5-star rated footwear for the prevention of slips in the workplace. It will be the largest trial of its kind to date. Primary outcome data on the number of slips experienced will be collected weekly via SMS. This is a novel and efficient method of collecting this data. It is quicker, less burdensome and cheaper than participants posting a questionnaire back and, due to the regularity of data collection, minimises the chance of recall bias.

Trial status

Recruitment and follow-up are in progress. The pilot phase of the trial has passed successfully, and the trial has continued seamlessly with no change to the sample size. Recruitment to the study began in March 2017 and will continue until approximately summer 2018. Participants will continue to be followed-up until winter 2018.

Acknowledgments

The research team would like to thank the members of the Trial Steering/Data Monitoring and Ethics Committee: Professor Avril Drummond, Dr Farina Hashmi, Mr Mark Thomas, Kate Chapman and Isabelle Smith for their advice, overseeing the study and reviewing adverse event data. The authors also extend very grateful thanks to the Patient Involvement Group, the study participants and members of the

For peer review only - http://bmjopen.bmj.com/site/about/guidelines.xhtml

Research and Development team for their help with the day-to-day running of the study and to Kevin Hallas from the HSE for his initial work on the study.

Author Contributions

DT, SC, CF, GF, CH, ML, MZ HI-S, LG, and RCB were either applicants on the NIHR PHR funding application or helped to refine the protocol. SC wrote the first draft of this article with input from CF and RCB. All authors read and approved the final manuscript.

Funding

This study is funded by the National Institute for Health Research (NIHR) Public Health Research (PHR; Project Reference Number 15/05/28) and the Health and Safety Executive. The views expressed are those of the author(s) and not necessarily those of the NHS, the NIHR, the Department of Health or the Health and Safety Executive. The University of York is the study sponsor and has legal responsibility for the initiation and management of the study: Sponsor representative Mr Michael Barber, Research and Enterprise Directorate, Innovation Office, York Science Park, Heslington, York UK YO10 5DG.

Competing Interest

Catherine Hewitt declares membership of the HTA Commissioning board. The NIHR grant application included some salary costs for SC, CF, GF, CH, ML, MZ, HI-S, LG, RC-B and DT. The HSE grant included some salary costs for ML, GF and MZ. GF, MZ, and ML declare that the HSE run the GRIP rating scheme, which was used to identify the footwear/manufacture to be used in the trial and Shoes for Crews paid the HSE for testing of footwear under the Grip rating scheme. No other competing interests are declared.

Ethics approval

The study protocol (version 4, 03.08.2017) including the original application and subsequent amendments (as required) received a favourable ethical opinion from the University of York, Department of Health Sciences Research Governance Committee, and, NHS Trust Research and Development at recruiting sites as well as Health Research Authority approval.

Peer review

This study was peer reviewed for ethical and funding approval prior to submission.

Data sharing statement

For peer review only - http://bmjopen.bmj.com/site/about/guidelines.xhtml

Requests to access SSHeW data can be made to the corresponding author once the trial is complete and the trial results published, and will be considered on a case-by-case basis by the Trial Management Group. All data requests will be managed according to York Trials Unit, University of York processes and procedures.

The Office for National Statistics is the provider of the Labour Force Survey data. The analysis of these data presented in this publication is the sole responsibility of the Health and Safety Executive.

REFERENCES

1. Health and Safety Executive. Kind of accident in Great Britain. http://www.hse.gov.uk/statistics/causinj/index.htm2017 [accessed 28.02.2018].
2. Labour Force Survey - Self-reported work-related ill health and workplace injuries: Index of LFS tables. http://www.hse.gov.uk/statistics/lfs/index.htm: Health and Safety Executive 2017.
3. Verma SK, Chang WR, Courtney TK, *al e*. A prospective study of floor surface, shoes, floor cleaning and slipping in US limited-service restaurant workers Occupational Environmental Medicine 2011;**68**:279-85.
4. Jensen OC, Laursen HL. Reduction in slips, trips and falls and better comfort by using new anti-slipping boots in fishing. *International Journal of Injury Control and Safety Promotion* 2010;**1**(18):85-7DOI: 10.1080/17457300.2010.487156.
5. Hallas K, Thorpe S, Liddle M, *et* al. The Role of Safety Footwear in Slipping Accidents. The 17th IEA World Congress on Ergonomics; Beijing,2009.
6. Blanchette MG, Powers CM. Validity and Accuracy of a Slip Resistance Measurement Protocol for the Assessment of Slip Potential. *American Society of Biomechanics* 2011 http://www.asbweb.org/conferences/2011/pdf/38.pdf [accessed 08/02/2013]. .
7. Patton M. Qualitative Evaluation and Research Methods 2nd ed: Newbury Park, CA: Sage; 1990
8. Devlin NJ, Shah KK, Feng Y *et al*. Valuing health-related quality of life: An EQ-5D-5L value set for England. *Health Economics*. 2017;**27**(1):7-22 doi:10.1002/hec.3564.
9. Silverman D. Interpreting Qualitative Data: Methods for Analyzing Talk, Text and Interaction: London: Sage 1993
10. Mays N, Pope C. Rigour and qualitative research. *BMJ* 1995;**311**:109-12.
11. Farmer T, Robinson K, Elliott SJ, *et al*. Developing and Implementing a Triangulation Protocol for Qualitative Health Research. *Qualitataive Health Research* 2006;**16**(3):377-94.
12. O'Cathain A, Murphy E, Nicholl J. Three techniques for integrating data in mixed methods studies. *BMJ*. 2010;341. doi: 10.1136/bmj.c4587.

Figure legend

Figure 1 Flow chart of participants through the pilot phase of the trial and recruitment to the SSHeW main trial.

For peer review only - http://bmjopen.bmj.com/site/about/guidelines.xhtml

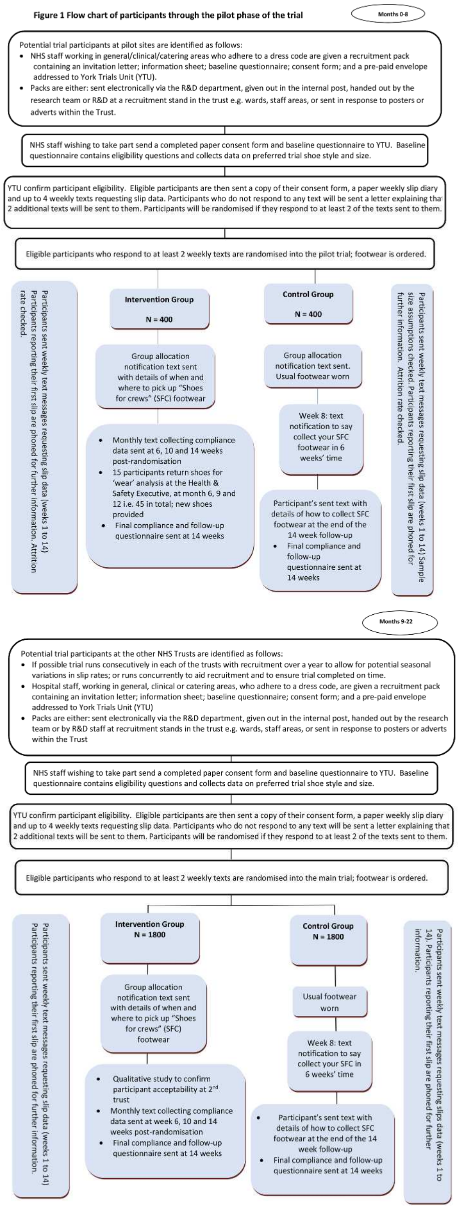

206x541mm (300 x 300 DPI)
