# Supplementary material for: SSHeW study protocol: does slip resistant footwear reduce slips among healthcare workers? A randomised controlled trial
Source: BMJ Open. 2018 Nov 15;8(11):e026023. doi: 10.1136/bmjopen-2018-026023 (PMC6252757; doi:10.1136/bmjopen-2018-026023)
Supplement: Reviewer comments [file bmjopen-2018-026023.reviewer_comments.pdf]

PEER REVIEW HISTORY

BMJ Open publishes all reviews undertaken for accepted manuscripts. Reviewers are asked to complete a checklist review form (**http://bmjopen.bmj.com/site/about/resources/checklist.pdf**) and are provided with free text boxes to elaborate on their assessment. These free text comments are reproduced below.

ARTICLE DETAILS

**TITLE (PROVISIONAL)**	The SSHeW study protocol: Does slip resistant footwear reduce slips among healthcare workers? A randomised controlled trial.
**AUTHORS**	Cockayne, Sarah; Fairhurst, Caroline; Frost, Gillian; Hewitt, Catherine; Liddle, Mark; Zand, Michael; Iles-Smith, Heather; Green, Lorraine; Cunningham-Burley, Rachel; Torgerson, David

This article was not externally reviewed at BMJ Open. Protocols that have been independently assessed prior to submission to BMJ Open are usually fast-tracked to publication on the grounds that further substantial changes will not be possible. This independent assessment will usually be external, independent review for both a substantial grant award from a non-commercial or government funder and ethics approval.
